# Emergence of a Thrombospondin Superfamily at the Origin of Metazoans

**DOI:** 10.1093/molbev/msz060

**Published:** 2019-03-13

**Authors:** Deborah K Shoemark, Berenice Ziegler, Hiroshi Watanabe, Jennifer Strompen, Richard P Tucker, Suat Özbek, Josephine C Adams

**Affiliations:** 1 School of Biochemistry, University of Bristol, Bristol, United Kingdom; 2 Centre for Organismal Studies, Department of Molecular Evolution and Genomics, University of Heidelberg, Heidelberg, Germany; 3 Evolutionary Neurobiology Unit, Okinawa Institute of Science and Technology Graduate University, Okinawa, Japan; 4 Department of Cell Biology and Human Anatomy, University of California at Davis, Davis, CA

**Keywords:** multicellularity, metazoa, extracellular matrix, regeneration, protein domains

## Abstract

Extracellular matrix (ECM) is considered central to the evolution of metazoan multicellularity; however, the repertoire of ECM proteins in nonbilaterians remains unclear. Thrombospondins (TSPs) are known to be well conserved from cnidarians to vertebrates, yet to date have been considered a unique family, principally studied for matricellular functions in vertebrates. Through searches utilizing the highly conserved C-terminal region of TSPs, we identify undisclosed new families of TSP-related proteins in metazoans, designated mega-TSP, sushi-TSP, and poriferan-TSP, each with a distinctive phylogenetic distribution. These proteins share the TSP C-terminal region domain architecture, as determined by domain composition and analysis of molecular models against known structures. Mega-TSPs, the only form identified in ctenophores, are typically >2,700 aa and are also characterized by N-terminal leucine-rich repeats and central cadherin/immunoglobulin domains. In cnidarians, which have a well-defined ECM, *Mega-TSP* was expressed throughout embryogenesis in *Nematostella vectensis*, with dynamic endodermal expression in larvae and primary polyps and widespread ectodermal expression in adult *Nematostella vectensis* and *Hydra magnipapillata* polyps. Hydra *Mega-TSP* was also expressed during regeneration and siRNA-silencing of *Mega-TSP* in *Hydra* caused specific blockade of head regeneration. Molecular phylogenetic analyses based on the conserved TSP C-terminal region identified each of the TSP-related groups to form clades distinct from the canonical TSPs. We discuss models for the evolution of the newly defined TSP superfamily by gene duplications, radiation, and gene losses from a debut in the last metazoan common ancestor. Together, the data provide new insight into the evolution of ECM and tissue organization in metazoans.

## Introduction

The extracellular matrix (ECM) of metazoans holds great fascination because of its roles in support of metazoan multicellularity and organismal complexity. In vertebrates, ECM is highly tissue specific and typically composed of around 150–300 proteins, along with carbohydrate components (Naba et al. 2012). Comparisons of the genome-predicted ECM proteins of bilaterian and nonbilaterian animals have demonstrated to date a suite of ECM proteins that are conserved between cnidarians and human. These include fibrillar-type collagens; associated proteins that are central to the production and assembly of collagen fibrils; laminin and collagen IV as core components of basement membranes, and thrombospondin (TSP) and SPARC/osteonectin as highly conserved matricellular glycoproteins (Exposito et al. 2010; Özbek et al. 2010; Bertrand et al. 2013; Fidler et al. 2017; Lommel et al. 2018). These ECM proteins are considered likely to have contributed to the origin of metazoan multicellularity and are deduced to have originated in or after the last metazoan common ancestor because equivalent ECM proteins are not encoded in choanoflagellates or *Capsaspora owczarzaki* that represent the closest unicellular relatives of metazoans (King et al. 2008; Fairclough et al. 2013; Suga et al. 2013; Williams et al. 2014; Brunet and King 2017).

The above research has emphasized the conservation of certain types of ECM proteins. Current genomic and transcriptomic resources make it feasible to explore another question of importance with regard to the evolution of complex multicellularity and the estimated rapid radiation of nonbilaterian phyla (Dohrmann and Wörheide 2017): the radiation and diversification of ECM proteins within early-diverging metazoan phyla. Here, we investigated this issue with regard to the TSPs, secreted glycoproteins that have wide pathophysiological significance in mammalian ECM and the pericellular environment (Adams and Lawler 2011; Murphy-Ullrich and Sage 2014; Stenina-Adognravi and Plow 2019). TSPs are multidomain, calcium-binding glycoproteins, many of which oligomerize cotranslationally as trimers (subgroup A) or pentamers (subgroup B) (Adams and Lawler 2011; Vincent et al. 2013). TSPs with domain architectures related to subgroup B have been identified in cnidarians and *Drosophila*, where they have roles in body axis maintenance and developmental ECM organization, respectively (Chanana et al. 2007; Tucker et al. 2013; Lommel, Strompen, Hellewell et al. 2018).

TSPs have many “domain relatives,” that is, proteins that have a single type of domain in common with TSPs. In particular, the epidermal growth factor (EGF)-like and thrombospondin type 1 domains (TSRs) are each widely represented in other categories of extracellular proteins (Tucker 2004; Wouters et al. 2005) and are also found outside the Metazoa (Adams and Lawler 2011). To date, the domain architecture of the C-terminal region of TSPs, that forms an integrated structural unit (comprising tandem repeated EGF-like domains, a series of calcium-binding TSP type 3 repeats and a C-terminal, L-type lectin domain), has been considered a unique signature of the TSPs (Kvansakul et al. 2004; Carlson et al. 2005).

A recent exploratory study of the TSPs of the anthozoan cnidarian, *Nematostella vectensis*, identified four transcribed paralogs. In phylogenetic trees based on the conserved TSP C-terminal region, one of these, Nv85341, has an unexpectedly close phylogenetic relationship with a thrombospondin of *Ciona intestinalis* termed TSP-DD (Tucker et al. 2013). Originally identified from expressed sequence tags, *C. intestinalis* TSP-DD was characterized by its apparent N-terminal discoidin-like domain (DD) and is secreted from cells as a monomer. At the time of its identification in 2010, protein sequence orthologs of TSP-DD were restricted to invertebrate deuterostomes (Bentley and Adams 2010).

The apparently anomalous identification of a possible TSP-DD-like polypeptide in *N. vectensis* led us to new investigations of early-diverging metazoans (cnidarians, poriferans, and ctenophores). We report here on previously undisclosed categories of TSP-related proteins, which we have designated mega-thrombospondin (mega-TSP), sushi-thrombospondin (sushi-TSP), and poriferan-TSP. All the predicted proteins are clearly related to TSPs by inclusion of the characteristic TSP C-terminal region domain architecture and differ in other domains and their phylogenetic distributions within the Metazoa. We present the first systematic evaluation of these proteins, their phylogenetic relationships with canonical TSPs, and the first analysis of biological function of a mega-TSP. These data illuminate the existence of an unappreciated TSP superfamily and lead to a new evolutionary scenario for the emergence of the canonical TSPs with implications for understanding of early metazoan evolution.

## Results

### Identification of New Categories of Conserved TSP-Related Proteins

Comparative genomic and transcriptomic searches were carried out initially with the predicted partial protein sequence of *N. vectensis* TSP85341 (Nv85341) and then with other representative TSPs. These led to the identification of further predicted TSP-related protein sequences in multiple cnidarians. Because some of these sequences are predicted as much longer polypeptides than a canonical TSP (e.g., 2,827 aa for XP_012565470 of *Hydra vulgaris*, whereas canonical TSPs range from 840 to 1,152 aa), the complete nucleotide sequence of the open reading frame (ORF) of Nv85341 was obtained by DNA sequencing from cDNA prepared from RNA from 2-month-old juvenile *N. vectensis* polyps. Similarly, a related ORF of *Hydra magnipapillata* (seq379420, XM_002157707) was confirmed and extended from a transcriptome database (Hydra 2.0 Web Portal, https://research.nhgri.nih.gov/hydra/; last accessed October 2018). The full ORF has 99% identity to XP_012565470 of *H. vulgaris*. The complete *N. vectensis* and *H. magnipapillata* protein sequences and closely related partial sequences from other cnidarians were then used to query genomic and transcriptomic databases at NCBI and other repositories, which led to identification of further categories of proteins.

The most frequently identified type of TSP-related protein was conserved in multiple metazoan phyla from ctenophores to basal chordates, yet was not identified in ecdysozoans (arthropods and nematodes) or craniates (hagfish, lamprey and jawed vertebrates). We designated these proteins “mega-thrombospondins” (mega-TSPs; MT) because of their very large size (typically >2,700 aa) and because the discoidin domain is not present in the earliest emerging forms (see below). In most species, a single mega-TSP was identified; some species encode two distinct gene products (table 1*A*). We also identified a separate category of TSP-related protein that contains repeated sushi domains near the N-terminus; this was designated “sushi-thrombospondin” (sushi-TSP; ST). The sushi-TSP group was identified (as of February 2019) to be encoded in poriferans of classes Calcarea and Homoscleromorpha and cnidarians of class Anthozoa, orders Scleractinia and Actiniaria (table 1*B*). A distinct form of TSP-related protein, containing multiple tandem TSRs, was identified only in poriferans and designated as “poriferan-TSP” (PT) (table 1*C*). For all three categories of TSP-related protein, evidence of transcription was obtained from expressed sequence tag and/or transcriptome databases, with some ORFs being predicted directly from transcriptome databases. An additional form of TSP, more closely related in domain organization to pentameric TSPs but lacking a distinct N-terminal domain or coiled-coil domain, was identified uniquely from the transcriptome of the calcareous sponge *Sycon ciliatum* (table 2).

**Table 1.** Phylogenetic Distribution of Mega-TSPs (*A*) Sushi-TSPs (*B*), and Poriferan-TSPs (*C*) Identified in this Study.

**Protein**/Phylum/*Species*	Accession Number (notes)	Length (aa)	Code Name
***A*. Mega-TSP (MT)**			
Ctenophora			
*Mnemiopsis leidyi*	MLRB34227 (sv)	2,323	MlMT
*Pleurobrachia bachei*	sb3464599(p), sb3460694(p)	nd	PbMT
*Homophoria californensis*	GGLO01050054(p)	2,549	HcaMT
Porifera			
*Leucosolenia complicata*	lcpid9057^a^	2,260	LcMT
*Sycon ciliatum*	scpid2291^a^	2,470	ScMT
Placozoa			
*Trichoplax adhaerens*	jgi|Triad1|51999|fgeneshTA2_pg.C_scaffold_1000746	2,494	TaMT
Cnidaria			
*Acropora digitifera*	XP_015754070(p)	1,623	AdMT
*Exaiptasia pallida*	KXJ17623.1	2,844	EpMT
*Hydra magnipapillata*	GAOL010126196.1(plus extension)	2,827	HmMT
*Nematostella vectensis*	MF962901	2,804	NvMT
*Porites australiensis*	FX462330.1	2,802	PaMT
Orthonectida			
*Intoshia linei*	OAF66671.1 (p)^b^	1,860	IlMT
Annelida			
*Capitella tellata*	JGIprotein221847(p)	813	CtMT
*Streblospio benedicti*	GDBG01124149 (p)	1,021	SbMT
Nemertea			
*Notospermus geniculatus*	g32562^b^	1,884	NgMT
Brachiozoa			
*Phoronis australis*	g6824^b^	2,821	PauMT
*Lingula anatina*	XP_013404445.1	3,277	LaMT
Mollusca			
*Crassostrea gigas*	XP_011414789.1	2,910	CgMT
*Pinctada fucata*	Pfu_aug1.0_1956.1_29913(p)^b^	1,535	PfMT
*Villosa lienosa*	JR504715	2,774	VlMT1
	JR505453	3,210	VlMT2
Echinodermata			
*Acanthaster planci*	XP_022095459	2,889	ApMT
*Asterias amurensis*	GAVJ010151310(p)	1,177	AaMT
*Ophiocoma echinata*	GAUQ01107960	2,366	OeMT
*Stronglyocentrotus purpurata*	XP_011666918, XP_011670237.1 (composite)	3,273^c^	SpMT
Chordata/Hemichordata			
*Saccoglossus kowalevskii*	XP_006823110(p)	1,931	SkMT
*Ptychodera flava*	GDGM01096791	2,920	PflMT
Chordata/Cephalochordata			
*Branchiostoma floridae*	XP_002595073(p)	1,931	BfMT1
	XP_002595072(p)	2,095	BfMT2
*Branchiostoma belcheri*	XP_019635900	2,609	BbMT
Chordata/Urochordata*Ciona intestinalis*	XP_002121519	3,020	CiMT
***B*. Sushi-TSP (ST)**			
Porifera			
*Leucosolenia complicata*	lcpid8282(p)^a^	1,268	LcST
*Oscarella carmela*	m162353^a^	1,355	OcST
*Sycon ciliatum*	scpid30246(p)^a^	1,143	ScST
Cnidaria			
*Acropora cervicornis*	GASU01084919(p)	1,207	AcST
*Orbicella faveolata*	XP_020606751	1,211	OfST
*Stylophora pistillata*	GARY01021319(p)	538	SpiST
*Nematostella vectensis*	JGINemve22035(p), NvERTx4150652	1,214	NvST
***C*. Poriferan-TSP (PT)**			
Porifera			
Demospongiae			
*Amphimedon queenslandica*	XP_019856517	1,090	AqPT
*Ephydatia muelleri*	m.239952^a^	1,032	EmPT
*Haliclona amboinensis*	c55984_g3_i1|mm.6896^a^	1,082	HaPT
*Halisarca caerulea*	GFSI01014595.1	864	HcPT
*Haliclona tubifera*	GFAV01006530/m.11915	1,200	HtPT
*Xestospongia testudinaria*	scaffold3224-augustus-gene-0.19^a^	1,572	XtPT
Calcarea			
*Leucosolenia complicata*	lcpid36553(p)^a^	1,045	LcPT
*Sycon ciliatum*	scpid12552(p)^a^	1,645	ScPT
Homoscleromorpha			
*Oscarella carmela*	comp38400_c0_seq2: 3-3362^a^	1,119	OcPT

Note.—Representative species are included for the phyla in which these TSP-related sequences were identified (BLAST *e*-values <1e-100). Accession numbers are from GenBank (proteins or TSA) unless otherwise indicated. Where two near-identical ORFs were reported from the same genome scaffold, or splice variants (sv) were detected, the longest ORF is listed. JGI: From JGI. NvERTx refers to http://ircan.unice.fr/ER/ER_plotter/home. Key: (p), partial sequence, N-terminus missing.

a From Compagen database.

b From OIST Marine Genomics genome browser.

c Estimated length from composite predicted polypeptide sequence.

**Table 2.** Complete List of TSP Superfamily Proteins and Additional Candidate TSP-Related Proteins Identified in Poriferans.

Poriferan Class/Species	Accession Code (GenBank or Compagen)				Notes
	Mega-TSP	Sushi-TSP	Poriferan-TSP	Other TSP/Possible TSP	
Demospongiae *Amphimedon queenslandica*	—	—	XP_019856517.1	—	XP_019856518.1 has the same sequence, 4 aa shorter.
*Ephydatia muelleri*	—	—	m.239952	m.78242 (p)	m.78242 is partial with 1xTSR, 3x EGF and T3.
*Haliclona amboinensis*	—	—	c55984_g3_i1|mm.6896	c39382_g2_i1|m.11684 (p)	c39382 is partial with T3 and 1x ConA.
*Halisarca caerulea*	—	—	GFSI01014595.1	—	
*Haliclona tubifera*	—	—	GFAV01006530/m.11915	—	
*Xestospongia testudinaria*	—	—	scaffold3224-augustus-gene-0.19	—	
Calcerea *Leucosolenia complicata*	lcpid9057lcpid45863 (p)	lcpid8282	lcpid36553 (p)	—	lcpid8493 same as 9057. lcpid8283 same as 8282. lcpid45863 is partial with 4xEGF, T3, 2xConA. lcpid36553 is partial with 6 xTSR, 3xEGF, T3, 1xConA.
*Sycon ciliatum*	scpid2291	scpid30246	scpid12552 (p) scpid43715 (p)	scpid23599	scpid26273 is the same sequence as sc30246. Scpid12552 and 43715 are partials, start at TSR.
Homoscleromorpha *Oscarella carmela*	—	m.162353	m.144001	m.297796 (p)	m.2997796 is partial, with T3 and 3 x ConA.

Note.—(p) indicates sequence is partial, incomplete at N-end. Identified from GenBank proteins or TSA, or via Compagen portal.

These studies also disclosed variation in the number of TSP-related proteins per species in poriferans, albeit that some identifications were uncertain due to ORFs that are incomplete for the N-terminus. The poriferans examined and the full sets of TSP-related proteins and candidate TSP-related proteins identified are summarized in table 2. On the basis of the transcriptomes, each species was found to encode multiple TSP-related proteins, yet only the calcerous species examined encode mega-TSP.

### Domain Architectures of Mega-TSP, Sushi-TSP, and Poriferan-TSP

**Fig. 1.**
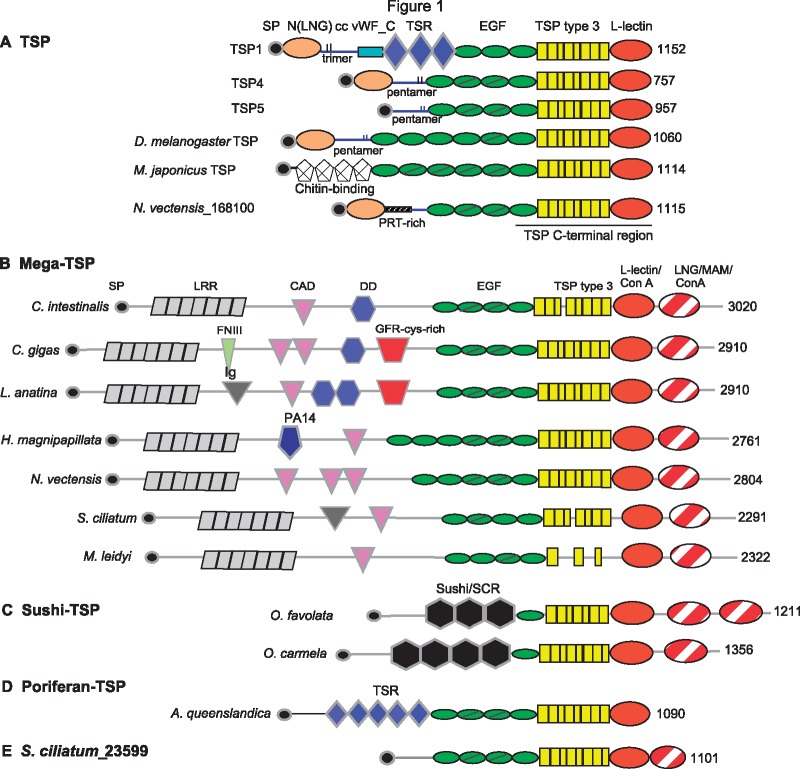
Schematic diagrams of the domain architectures of representative TSPs and TSP superfamily proteins. The length of each polypeptide is indicated at the righthand of each model. The animal groups and database identifiers for each polypeptide are given in table 1. Key: SP, signal peptide; LGN, laminin G-like N-terminal domain; cc, coiled coil; vWF_C, von Willebrand factor C domain; TSR, thrombospondin type 1 domain; EGF, epidermal growth factor-like domain; PRT, proline-arginine-threonine-rich region; LRRs, leucine-rich repeats; CAD, cadherin domain; DD, discoidin domain; FNIII, fibronectin type III domain; GFR-cys-rich, growth factor receptor cysteine-rich domain; Ig, immunoglobulin domain; and SCR, short consensus repeat.

The domain architecture of EGF-like domains, TSP type 3 repeats and a single L-type lectin-like domain (also sometimes annotated as “TSP-C” or “Concanavalin A-like” (Con A-like) domain at the C-terminus is a defining feature of canonical TSPs (fig. 1*A*; the animal groups to which these species belong are given in table 1). Domain analysis of full-length sequences representative of the newly identified TSP-related proteins showed that each has a distinctive, characteristic domain architecture. Thus, all mega-TSPs identified have the conserved features of a N-terminal secretory signal peptide; an extensive region (around 1,000 aa) predicted as multiple, tandem repeated leucine-rich repeats (LRRs); a central region of variable length and domain composition in which one or more cadherin domains are a well-conserved feature; a canonical TSP C-terminal region of 900–1,000 aa, followed by a second globular domain at the C-terminus that is variably annotated as “TSP-C,” “ConA-like,” or “MAM” (meprin, A-5 protein, and receptor protein-tyrosine phosphatase mu [Beckmann and Bork 1993]), and a terminal, nonconserved sequence (fig. 1*B*). The multiple annotations of the ConA-like domains are not surprising given that the Con A domain has a β-jellyroll structure that is shared by L-type lectins, MAM domain proteins and several other domain groups including the laminin G domain (InterPro [IPR] 013320 [Williams and Westhead 2002]). None of the mega-TSP protein sequences contained a coiled-coil domain, whereas most canonical TSPs oligomerize by the action of a coiled-coil domain (Bentley and Adams 2010; Vincent et al. 2013) (fig. 1*A* and *B*).

In mega-TSPs from ctenophores, the central region includes a single cadherin domain (fig. 1*B*). In mega-TSPs from sponges, the region also includes the immunoglobulin-like domain and, in *Leucosolenia complicata* mega-TSP, a “tyrosine kinase ephrin A/B receptor-like” domain (IPR01641) in addition to a cadherin-like domain: it can be noted that immunoglobulin-like and cadherin domains belong to the same “Greek key,” beta-sandwich fold group (Hutchinson and Thornton 1993). All cnidarian mega-TSPs examined include one or more cadherin domains in the central region, and *H. magnipapillata* mega-TSP (HmMT) also includes a beta-barrel PA14 domain (InterPro011658) (fig. 1*B*). In mega-TSPs from bilaterians, the central region also typically includes a fibronectin type III domain (also a member of the Greek key fold group), one or more F5_F8 or DD domains, and a growth factor receptor cysteine-rich domain (IPR009030; a fold related to the “tyrosine kinase ephrin A/B receptor-like domain”) (fig. 1*B*). Thus, the DD is not intrinsic to the mega-TSPs but appears as a bilaterian-specific domain addition.

Sushi-TSPs are shorter proteins (around 1,000–1,300 aa) that also lack a coiled-coil domain and are distinguished by the presence of three or more tandem sushi/short consensus repeat domains in the N-terminal region and a single EGF-like domain. In common with the mega-TSPs, the sushi-TSP of the sponge, *Oscarella carmela*, has tandem TSP-C/Con A-like and Con A/MAM-like domains proximal to the C-terminus, whereas sushi-TSPs from *Orbicella faveolata* and *Acropora digitifera* (both cnidarians) include three Con A-like domains at the C-terminus (examples in fig. 1*C*).

The poriferan-TSPs, identified in sponges from classes Demospongia, Calcarea, and Homoscleromorpha, have a closer resemblance of domain composition to canonical TSPs, but lack a laminin-G-like N-terminal domain or a coiled-coil domain and contain extended sets of tandem TSR domains (only three TSR are present in TSP1 and TSP2 of vertebrates). The full-length sequences identified from the demosponges *Amphimedon queenslandica* (Srivastava et al. 2010), *Haliclona tubifera* (GenBank transcriptome GFAV00000000.1), and *Xestospongia testudinaria* (Ryu et al. 2016) (table 1), each also contain an uncharacterized region of about 130 aa after the signal peptide, followed by five or six TSR domains, four EGF-like domains, tandem TSP type 3 repeats and a single, Con A-like, C-terminal domain (fig. 1*D*). A unique form of TSP-related protein was also identified in the calcareous sponge *S. ciliatum*, in which the secretory signal peptide is followed by an unrelated uncharacterized region, four EGF-like domains, TSP type 3 repeats and two C-terminal, ConA-like domains (table 2 and fig. 1*E*).

Because none of the TSP-related proteins were identified in nonmetazoans, we examined the representation of their component domains in other eukaryotes, with emphasis on the lineages of protists most closely related to Metazoa (supplementary fig. 1*A*, Supplementary Material online). With the exceptions of TSP-N and TSP-C domains and the TSP type 3 repeats, all other major conserved domains of TSP superfamily members are characterized in the InterPro database as conserved in other unikont lineages. The structural fold-family of the TSP-N and TSP-C domains, as represented by LN-G and Con-A domains, is widely represented in unikonts (supplementary fig. 1*B*, Supplementary Material online). We also searched the genome-predicted proteins of relevant protist species, with emphasis on the closest relatives of metazoans, for the major conserved domains of TSP superfamily members. This approach yielded similar data, although the vWF_C domain was not identified in these species. The most widely conserved domains were the LRR, EGF-like, and sushi domains: all the other domains showed more limited phylogenetic representation yet were all represented in choanoflagellates (supplementary fig. 1*C*, Supplementary Material online). Thus, the separate domains of TSP superfamily members evolved before the Metazoa.

### Molecular Modeling of LRRs and TSP C-Terminal Region

**Fig. 2.**
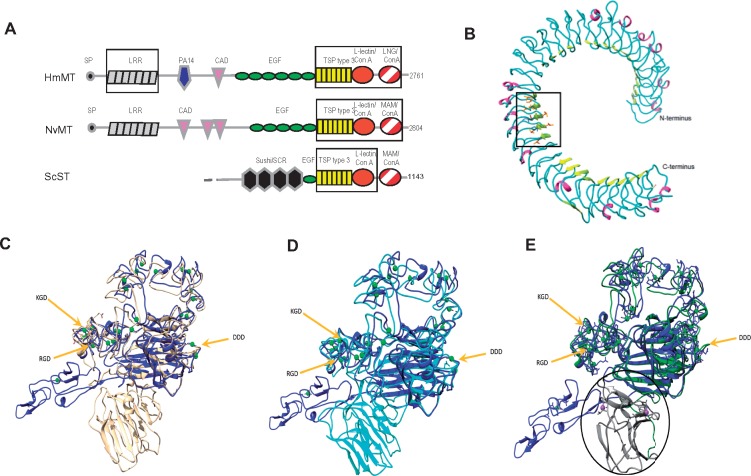
Molecular modeling of the LRRs and C-terminal region from representative cnidarian mega-TSPs and a sponge sushi-TSP. (*A*) Domain schematics of the TSP superfamily proteins used for modeling. Boxes indicate the regions that were modeled. Key as in figure 1. (*B*) Model of the extended N-terminal region of HmMT, with ribbon coloured according to secondary structure (beta strand, yellow; alpha helix, magenta, and loops, cyan). The boxed area indicates how the LRRs align in the model. Within the box, leucine side chains are represented by orange sticks and neighboring isoleucines in blue. (*C*–*E*) Models of the C-terminal region from HmMT (beige tube in *C*), NvMT (cyan tube in *D*), and ScST (green tube in *E*), each overlaid with the C-terminal structure of TSP1, PDB1YO8 (blue tube in each panel). The positions of functional motifs of TSPs are arrowed in each panel.

To examine whether the predicted LRRs and TSP-like C-terminal regions of the TSP-related proteins conformed to known structures, molecular modeling was carried out on the relevant sequence regions of mega-TSPs and sushi-TSP from cnidarians and a sponge, respectively (fig. 2*A*). HmMT was used to model the predicted LRR region of 863 aa. This region could be modeled as a set of tandem LRR with good correspondence to known structures of tandem LRR, with the inner face characterized by beta-sheets and a predominance of loop structures on the outer face (fig. 2*B*).

The HmMT C-terminal region sequence shared 42% identity with the C-terminal region of TSP5, on which structure (PDB 3FBY, Tan et al. 2009), the model was based. The second ConA-like domain shared 21% identity with the structure of PDB 4DQA and the model was based on this structure. The separate alignments of HmMT with 3FBY and 4DQA overlapped in part and were in agreement in the overlap region, which added support to the modeled conformation of the two ConA-like domains with respect to each other (fig. 2*C*). The short alpha-helical C-terminal extension from the ConA domain of the 3FBY crystal structure provided a reasonable point of attachment for modeling the second ConA domain in HmMT, allowing sufficient space to comfortably accommodate the model of the second ConA-like domain. The C-terminal region of *N. vectensis* mega-TSP (NvMT) was also modeled on PDB 3FBY (of 46% identity) and the second Con A-like domain on PDB 4DQA (of 21% identity). Again, the model had good correspondence to the TSP domain structure (fig. 2*D*). There was no sequence overlap in these alignments, so the orientation between the two Con A-like domains is less well supported.

For the model of the C-terminal region of *S. ciliatum* sushi-TSP (ScST), the type 3 repeats and first ConA-like domain had 49% sequence identity with 3FBY and corresponded well with this structure (fig. 2*E*). However, the second ConA-like domain could not be modeled due to low identity with the structures in the RCSB structural databank. The sequence identity of the second ConA-like domain between HmMT and NvMT is 31%, between HmMT and ScST is 20.1%, and for NvMT and ScST is 22%. Thus, these domains of mega-TSPs are more similar to each other than they are to the available structures. In view of the possible conservation of a second ConA-like domain of ScST, the 4DQA structure is shown placed in an analogous position (fig. 2*E*). Overall, the models of the TSP-like C-terminal regions corresponded very well to known TSP structures, supporting the relationship with canonical TSPs.

Procheck (Laskowski et al. 1993) was used to evaluate the “protein-like” quality of the models with respect to phi-psi angles for residue types in Ramachandran space, bond lengths, and other parameters. This revealed that 98.6% of residues 108–971 of HmMT in the LRR model and 99.6% of the residues in the NvMT C-terminal region model were in favored or allowed regions of Ramachandran space and the quality of the models’ geometry was better than a 2–2.5-Å structure. The extended N-terminal LRR of the HmMT model showed adequate correspondence given that the sequence homology with templates was low and discontinuous. For the modeled C-terminal regions, the conservation of disulfide bridges with the TSP C-terminal structures provided additional confidence in self-consistency (table 3).

**Table 3.** Protein Characteristics from Molecular Modeling Predictions.

Model	Number of Residues	% Optimal	% Allowed	% Generously Allowed	% Disallowed
HmMT LRR	863	63	32	3.4	1.4
HmMT C-term	673	65	31.4	2.5	1.2
NvMT C-term	671	69.4	28	1.6	1.1
ScST C-term	534	82.5	15.1	1.9	0.4

Note.—Models were analyzed with Procheck.

### Partial Conservation of Functional Motifs of TSPs in TSP Superfamily Proteins

Several peptide motifs have been identified within the C-terminal regions of canonical TSPs that have important roles in cell or ECM interactions (arrowed in fig. 2*C*–*E*). The possible conservation of these motifs was first examined by multiple sequence alignment of the C-terminal regions. In TSP1, an Arginine-glycine-aspeactic acid (RGD) motif in type 3 repeat 7 coordinates a calcium ion and can bind integrins αvβ3 and αIIbβ3 (Lawler and Hynes 1989; Kvansakul et al. 2004). A RGD motif at the equivalent position was conserved in subgroup A TSPs of chordates, *Marsupenaeus japonicus* TSP and a single mega-TSP (supplementary fig. 2*A*, Supplementary Material online). A Lysine-glycine-aspartic acid (KGD) motif in type 3 repeat 6 of TSP1 and equivalently positioned RGD in TSP2, also coordinate a calcium ion (Kvansakul et al. 2004; Carlson et al. 2005). Equivalent KGD or RGD motifs are conserved in some other TSPs, some poriferan-TSPs and a single mega-TSP (supplementary fig. 2*B*, Supplementary Material online). Within the L-lectin/Con A-like domain of TSPs, a highly conserved DDD motif coordinates calcium ions and controls deposition of trimeric or pentameric TSPs into cell-derived ECM (Kim, Christofidou et al. 2015). The DDD motif is conserved in the first L-lectin/Con A domain of most sushi-TSPs and some poriferan-TSPs, but not in mega-TSPs (supplementary fig. 2*C*, Supplementary Material online). The second, Con A-like domain of *Orbicella faveolata* sushi-TSP also contains a DDD motif but this did not align with the conserved position. The absence of the DDD motif from the Con A-like domains of mega-TSPs implies that these proteins do not share the mechanism for ECM retention identified for canonical TSPs.

**Fig. 3.**
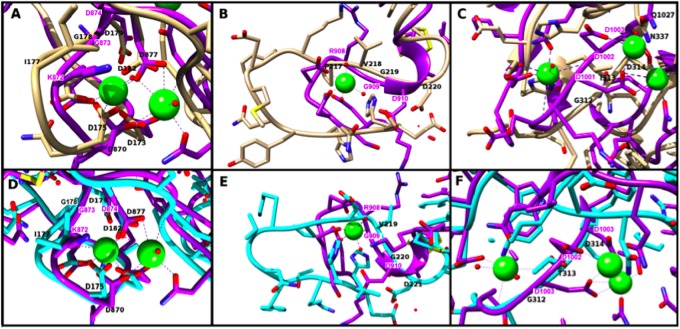
Modeling of *Hydra magnipapillata* and NvMTs against functional motifs of TSP1. In (*A*–*C*), the HmMT model (beige tube) is overlaid on the crystal structure 1UX6 (purple ribbon). (*D*–*F*) The corresponding regions of the NvMT model (cyan tube) overlaid on 1UX6 (purple ribbon). In all panels, calcium ions are shown as green balls. (*A* and *D*) The overlays around the KGD motif in the type 3 repeats. The KGD motif (K872, G873, and D874) of 1UX6 is overlaid by I177, G178, and D179 of the HmMT model (*A*), or I177, G178, and D179, of the NvMT model (*D*). (*B* and *E*) Overlays of the structure around the RGD motif in the type 3 repeats. The RGD site of 1UX6 (R908, G909, and D910) overlays with P217, V218, and G219 of the HmMT model (*B*), or V219, G220, and D221 of the NvMT model (*E*). (*C* and *F*) Overlays around the DDD motif in the first L-lectin/Con A-like domain of the mega-TSPs. The DDD site of 1UX6 (D1001, D1002, and D1003) is overlaid by G312, I313, and D314 of the HmMT model (*E*), or G312, T313, and D314 of the NvMT model (*F*).

To deeply investigate the locations of these motifs and their potential for calcium coordination, relevant regions of the C-terminal molecular models were compared in detail to the TSP1 C-terminal structure PDB 1UX6 (Kvansakul et al. 2004). At the KGD motif of 1UX6 (1UX6 positions 872–874), the corresponding residues of the overlaid HmMT model (positions 177–179 of the model) are Isoleucine, glycine-aspartic acid (IGD) and the aspartate residues in this region of the model overlay those of 1UX6 (fig. 3*A*). In the overlay of NvMT, the residues corresponding to KGD (177–179 in the model) are also IGD and the aspartate positions (NvMT D171, D173, D175, D179, and D182) are entirely equivalent to those in the 1UX6 structure (fig. 3*D*). Thus, in both models, this site appears to provide sufficient negative charge to support calcium binding and it is predicted that calcium binding is retained (fig. 3*D*). Cysteines C181-C161 in the NvMT model also appeared to be conserved with the disulfide C876-C856 in 1UX6 which constrains the structure and may stabilize potential calcium-binding sites (Kvansakul et al. 2004).

Considering the RGD motif of 1UX6, the analogous residues in HmMT are PVG (residues 217–219 of the model). In the 1UX6 structure, these residues constrict the entrance to a short loop with D902, S903, D904, G905, and D906, and these three aspartates chelate a calcium ion. In the HmMT model, the analogous loop is extended and the negatively charged residues do not appear to be in favorable positions to support calcium binding (fig. 3*B*). In NvMT, the residues corresponding to RGD of 1UX6 are VGD (residues 219–221 of the model). Similar to the HmMT model, the associated loop is extended in comparison with the RGD-containing loop of 1UX6 (fig. 3*E*). Without a significant deviation from the fold predicted in the model, it is unlikely that the neighboring acidic residues such as E206 and E217 of NvMT can provide the necessary orientation of negative charge for coordination of a calcium ion. Thus, the models suggest that the negative charge in this region would be insufficient to support calcium binding.

With regard to the conserved DDD motif in the L-lectin/Con A-like domain of TSPs, the corresponding residues in HmMT are GID (aa312–314 of the model). Although D314 of HmMT overlays D1003 of 1UX6, N337, of HmMT overlays Q1027, and the HmMT model provides two glutamates (E311 and E336) in this vicinity, on balance, it appears that the required orientation of charges for calcium binding is unlikely to be achieved at the single aspartate residue (fig. 3*C*). In the NvMT model, the residues corresponding to DDD are GTD (NvMT aa312–314; shown in cyan in fig. 3*F*). Similarly, by inspection, this region appears unlikely to provide sufficient negative charge for calcium ion binding. In summary, Ca-binding is predicted to be conserved in type 3 repeat 6, but not at the RGD-equivalent site or in the Con A-like domains of mega-TSPs.

### Expression Pattern of Mega-TSP in *N. vectensis*

**Fig. 4.**
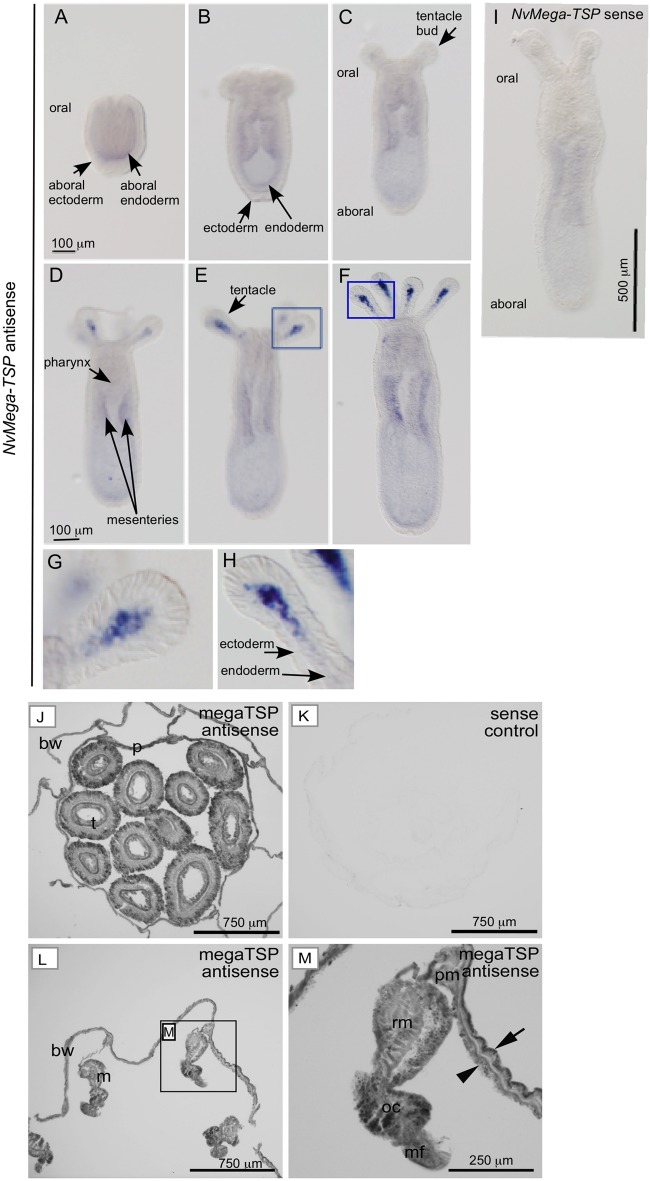
Expression of mega-TSP in *Nematostella vectensis*. (*A*–*F*) Representative whole-mount in situ hybridisation results obtained with an antisense *NvMT* probe on planula larva (*n* = 15) (*A*) or primary polyps (*n* = 19) (*B*–*F*). (*G*–*H*) Enlargements from boxed areas of (*E*–*F*) show details of *NvMT* expression in tentacles. (*I*) Representative image of adult *N. vectensis* probed with the *NvMT* sense probe. (*J*–*M*) Representative images of adult *N. vectensis* (*n* = 4) probed with the *NvMT* sense probe. (*J* and *K*) Cross sections of the pharynx of an animal that had retracted its tentacles just prior to fixation, hybridised with an antisense probe (*J*) or a sense probe (*K*). In (*J*), the signal concentrated in the ectoderm (arrowed). bw = body wall, p = pharynx, t = tentacle. (*L* and *M*) A cross section of the body wall and associated tissues, probed with an antisense probe. (*M*) Displays the boxed area from (*L*). Signal is stronger in the epidermis (arrow) than in the gastrodermis (arrowhead), and stronger in the ovarian cyst (oc) than elsewhere, along with clear signals in the retractor muscles (rm), parietal muscles (pm) and mesenteric filament (mf).

In view that mega-TSPs have the widest phylogenetic distribution of the newly identified superfamily members, we chose to focus on mega-TSPs for initial biological investigations. Cnidarians were chosen with regard to their well-studied biology and as the only early-diverging phylum in which canonical TSPs have been studied. *Nematostella vectensis* is well developed as a laboratory model for the anthozoan class of cnidarians (Layden et al. 2016). Tissue expression patterns of *NvMT* were examined by in situ hybridization at several life-cycle stages. In motile planula larvae (ca. 72-h postfertilization [pf]), the ectodermal layer was negative and the *NvMT* transcript was detected specifically as faint expression in the aboral endoderm (fig. 4*A*). In primary polyps, faint expression continued in the endoderm, but not in the pharynx (shown at 5d pf in fig. 4*B* and *C*). In more developed polyps, endodermal expression became more evident at the mesenteries and was most obvious in some cells in the endodermal layer at the tips of tentacles; this signal became stronger as the tentacles developed (shown at 7d pf in fig. 4*D*–*F*; boxed areas in fig. 4*E and F* are enlarged in fig. 4*G* and *H*). These patterns were not detected with an *NvMT* sense probe (fig. 4*I*). We also analyzed *NvMT* transcript expression from a transcriptomic database that includes timepoints from the earliest stages of embryogenesis (Warner et al. 2018). The timecourse indicated relatively high *NvMT* transcript levels in the earliest stages up to 12hpf (likely due to maternal transcripts), lower expression in blastulae, increasing expression throughout the gastrula (24h–48hpf) and planula (48h–120hpf) stages, and maintenance of higher expression in juveniles (from 120hpf onward) (supplementary fig. 3*A*, Supplementary Material online). The data on planula larvae and juveniles are in line with the in situ data.

In adult *N. vectensis* polyps, examined as transverse sections through several body regions, the *NvMT* transcript was detected most strongly in the ectodermal layer. This was particularly apparent in a cross section through the pharynx of an animal with retracted tentacles (fig. 4*J*, the sense control is shown in fig. 4*K*), whereas the signal in the body wall of the pharynx was weaker. Sections through the gastric region of the body column also revealed widespread expression in the body wall and associated tissues and higher expression in the ectoderm than the endoderm (fig. 4*L* and *M*). In internal tissues, transcript expression was higher in the ovarian cyst and mesenteric filament than in body wall muscles (fig. 4*M*). Analysis of a head regeneration transcriptome timecourse database (Warner et al. 2018) indicated that *NvMT* expression drops irregularly during initial wound healing and then progressively increases during later stages of tissue remodeling and tentacle regrowth (supplementary fig. 3*B*, Supplementary Material online).

### Expression Pattern and Functional Role of Mega-TSP in Head Regeneration of *Hydra*

**Fig. 5.**
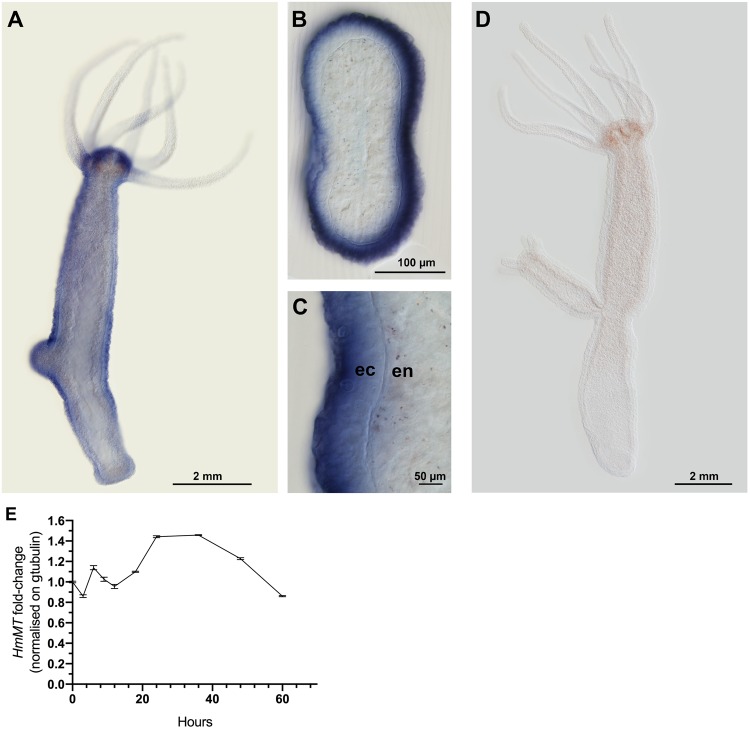
Expression of mega-TSP in *Hydra magnipapillata*. (*A*–*C*) In situ hybridization results obtained with an antisense LNA probe to *HmMT*; (*D*) a control staining with no LNA probe. (*A*) Representative expression pattern of *HmMT* in a whole-mounted, adult *Hydra magnipapillata* polyp. (*B*) Cross section of the gastric region. (*C*) Enlargement of cross section through the body wall to show the predominance of *HmMT* transcript in the ectoderm (ec) versus the endoderm (en). Arrow indicates the position of the mesoglea. (*D*) Representative image of adult *Hydra* stained without the Hm mega-TSP antisense LNA probe. Representative of 10 polyps examined. (*E*) Quantitative real-time PCR for *HmMT* transcript abundance, normalized against γ-tubulin, over the indicated timecourse of head regeneration, with 0 representing the time of transection. Each datapoint is the mean ± SD from two independent experiments, with RNA from 50 regenerating tips (each 10% of body length) per timepoint per experiment.

*Hydra* is a representative of the medusozoans and has been studied for over 200 years as an accessible animal with a simple body plan and high regenerative capacity (Chapman et al. 2010). The sequenced genome of *H. magnipapillata* encodes a single canonical TSP (Bentley and Adams 2010; Lommel, Strompen, Hellewell et al. 2018) and gene-silencing technology is well developed, therefore this species was chosen for examination of mega-TSP expression and function. Whole-mounts of *H. magnipapillata* polyps had striking expression of *HmMT* in the body wall with the exception of the tentacles. Signal intensity was highest around the hypostome, mouth, gastric, and budding regions and was weaker in the peduncle and basal disc (fig. 5*A*; a negative control is shown in fig. 5*D*). Cross sections through the gastric region emphasized the extensive and uniform signal within the body wall (fig. 5*B*). Higher power views clarified that *HmMT* was expressed preferentially in the outer, ectodermal cell layer of the body wall, with little or no signal detected in the endoderm (fig. 5*C*).

The elevated expression of *HmMT* in the head and budding region of Hydra suggested a possible function in head patterning or regeneration. Quantified real-time polymerase chain reaction (qPCR) of *HmMT* transcript during head regeneration after transection showed a small initial decrease, recovery to similar abundance between 6 and 18 h, and increased abundance between 24 and 48 h followed by a decline (fig. 5*E*). The 24–48-h period corresponds to the onset of assembly of a new head (MacWilliams 1983).

**Fig. 6.**
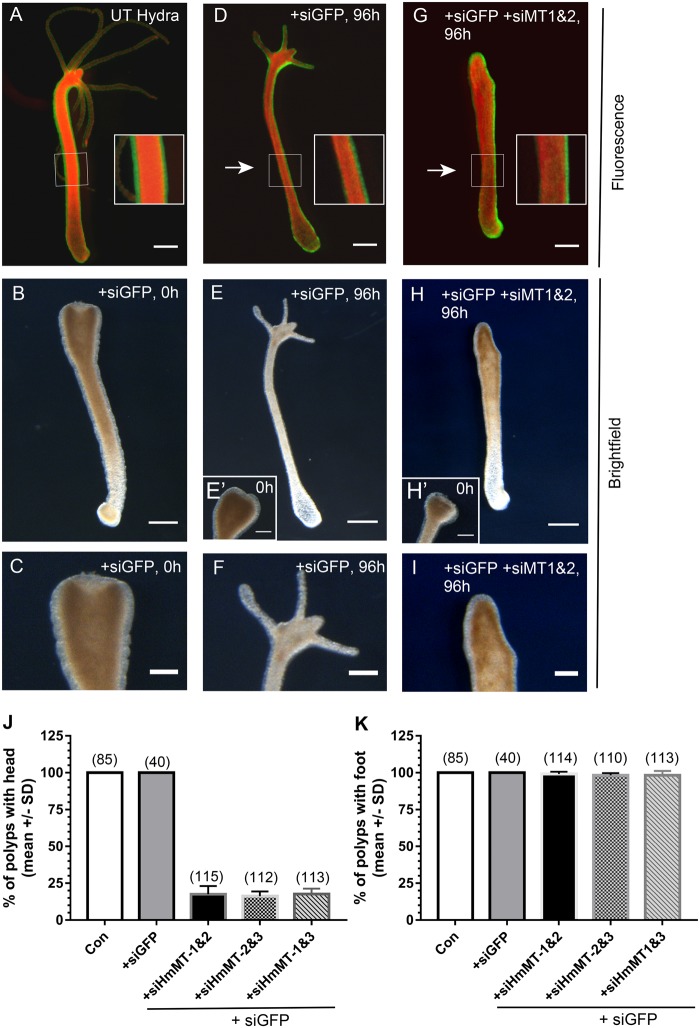
Head regeneration in hydra is inhibited by siRNA-silencing of *HmMT* expression. (*A*, *D*, and *G*) Merged fluorescence microscopy images of the GFP-ectoderm, RFP-endoderm chimeric animals; arrows indicate direction of electroporation. Insets show enlargements of the boxed areas to demonstrate loss of GFP signal, which appears one sided due to the asymmetric current applied during electroporation and the very strong expression of the GFP transgene. (*B*, *E*, and *H*) Corresponding brightfield views and (*C*, *F*, and *I*) enlarged brightfield views of head regions. (*A*) Untreated control polyp; (*B*) representative si*GFP*-electroporated polyp immediately after transection with no hypostome or tentacles; (*C*) enlarged view of transection site from (*B*). (*D* and *E*) Representative si*GFP*-treated polyp showing regeneration of hypostome and tentacles after 96 h. (*E*′) The transection site at 0 h; (*H*′) enlarged view of regenerated head at 96 h. (*G* and *H*) Representative polyp treated with si*GFP* and si*HmMT*1&2 lacks head regeneration at 96 h. (*H*′) The transection site at 0 h; (*I*) enlarged view of transection site at 96 h. In (*A*, *B*, *D*, *E*, *G*, and *H*) scale bars = 500 μm; in (*E*′, *H*′, *C*, *F*, and *I*) bars = 250 micron. (*J*, *K*) Quantification of head (*J*) or foot (*K*) regeneration at 96 h after transection of polyps and the indicated treatments. Each column represents the mean and bars the s.e.m. from three independent experiments. The total number of polyps analyzed per condition is stated above each column.

To test for a functional role of HmMT in regeneration, we analyzed the capacity for head or foot regeneration of polyps in which *HmMT* expression was silenced by electroporation of short interfering RNAs (siRNA). For these experiments, a transgenic Hydra line expressing green fluorescent protein (GFP) in ectodermal cells and red fluorescent protein (RFP) in endodermal cells (Carter et al. 2016) was used (fig. 6*A*), so that the effectiveness of knockdown could be monitored by applying si*GFP* together with si*HmMT*. In animals electroporated with si*GFP* only, the GFP signal was abolished in the ectodermal layer of the affected side of the polyp (compare fig. 6*A* with fig. 6*D*; insets show enlarged views of the body wall). Head regeneration of si*GFP* animals was completed normally by 96 h after transection (compare fig. 6*B*, *C*, and *E*′ at 0 h with fig. 6*E* and *F* at 96 h). Upon electroporation of si*HmMT*, head regeneration was almost totally inhibited even after 96 h (fig. 6*G–I*;fig. 6*H′* shows the transected polyp in fig. 6*H* at 0 h). The green patch of ectodermal cells covering the oral end of the nonregenerating polyp in figure 6*G* is explained by the continuous tissue flow from the midgastric region toward the head and tentacles that involves the mesoglea and epithelial cells (Aufschnaiter et al. 2011). The movement of GFP-positive cells toward the head region is likely slowed more sharply on the side closest to the electroporation source. The functional requirement for *HmMT* in head regeneration was reproducible with three different siRNAs to *HmMT* used in different pairwise combinations (fig. 6*J*). In contrast, none of the siRNAs blocked foot regeneration (fig. 6*K*). These data establish that HmMT has a significant role in head regeneration of *Hydra*.

### Phylogenetic Relationships of TSPs and TSP Superfamily Proteins

**Fig. 7.**
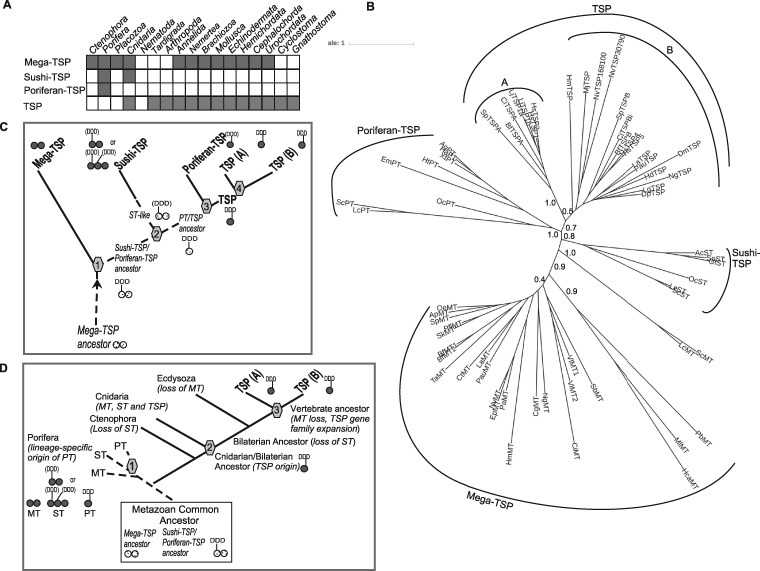
Phylogenetic analysis and models for the evolution of the TSP superfamily. (*A*) Summary of phylogenetic distribution of TSP superfamily proteins. See table 1 and Materials and Methods for underlying data. (*B*) Phylogenetic tree of TSP superfamily proteins based on the conserved domains of the C-terminal region. Sequences from 63 species were aligned in MUSCLE (1,048 positions) and the tree constructed in PhyML with 200 cycles of boot-strapping. The unrooted tree is presented with proportionate branch lengths. Boot-strap branch support values for the deepest branches are shown. Scale bar = substitutions/site. Codes for species names are as in table 1. (*C*) Model for the evolution of the TSP superfamily based on “ctenophores first” metazoan evolution. (*D*) Model for the evolution of the TSP superfamily based on “poriferans first” metazoan evolution. In (*C* and *D*), numbers refer to proposed gene duplication events. The circular diagrams indicate the number of Con A-like domains present in the different proteins and whether a DDD motif is present. Brackets indicate that the DDD motif is found in some but not all extant species orthologs. Light gray = hypothesized ancestral form, dark gray = extant form. See Discussion for details.

Our comparative genomics analysis established that mega-TSPs are the only TSP-related proteins present in ctenophores, whereas multiple forms are present in poriferans and cnidarians (fig. 7*A*). Indeed, mega-TSP, sushi-TSP, and poriferan-TSP are each represented by distinct gene transcripts in the calcareous sponge *S. ciliatum* (fig. 7*A* and table 2).

Next, molecular phylogenetic analyses were carried out on the relationships of the newly identified TSP-related proteins and the canonical TSPs. With regard to mega-TSPs and sushi-TSPs that have more than one Con A-like domain, an unrooted phylogenetic tree of individual Con A-like domains showed that all the “N-terminal” Con A-like domains (i.e., the Con A-like domain immediately after the type 3 repeats) are more closely related to each other than to any of the additional Con A-like domains (supplementary fig. 4, Supplementary Material online). Therefore, further phylogenetic analysis was based on the region comprising the last EGF-like domain, type 3 repeats and first Con A-like domain. Each category of sequences formed a distinct clade separate from the canonical TSPs. The tree topology as recovered by several methods placed sushi-TSPs closer to mega-TSPs and poriferan-TSPs closer to canonical TSPs (fig. 7*B*). Considerable sequence diversity was apparent (from branch lengths) within the poriferan TSPs and mega-TSPs. Within the canonical TSPs, as expected from prior studies, subgroup A TSPs formed a separate subclade from other TSPs (fig. 7*B*). Although TSP sequences from cnidarians to human were included, the TSP clade showed more limited evolutionary divergence than mega-TSPs or poriferan-TSPs, suggestive of a shorter evolutionary divergence time, and/or higher conservation/greater selection pressure.

## Discussion

Among the hundreds of proteins in the metazoan extracellular milieu, TSPs have been considered a unique form of matricellular and ECM protein. Here, we identify, by comparative genomics, domain composition and molecular modeling, multiple forms of TSP-related proteins (mega-TSP, sushi-TSP, and poriferan-TSP) that all share a domain region equivalent to the C-terminal region of TSPs. These identifications provide the first evidence for evolution of a TSP superfamily. Whereas secreted TSP-like proteins are not present in the close relatives of metazoans (Williams et al. 2014), we find the component domains are encoded, indicating a likely origin of the TSP superfamily as novel gene product(s) in the metazoan common ancestor. Within the Metazoa, one or more superfamily members are transcribed along with canonical TSPs in many species, yet all are absent from nematodes, and only canonical TSPs were identified in tardigrades and arthropods. This is in line with the distinctive ECM composition of cuticle-bearing nematodes and other ecdysozoans (Johnstone 2000). Mega-TSPs are encoded in many modern metazoan phyla, from Ctenophora to Chordata, but appear absent from the Ecdysozoa, tardigrades and vertebrates.

Potential molecular activities of mega-TSP and sushi-TSP were explored by analysis of molecular structures and motifs. The C-terminal regions of canonical TSPs have roles in binding cell surface receptors as well as other ECM proteins (Adams and Lawler 2011) and we speculate that the N-terminal, LRR region of mega-TSPs allows for additional extracellular protein–protein interactions at cell surfaces. LRR occur in many proteins such as the TOLL-like receptors that function in innate immune defense. These receptors bind to pathogen-associated proteins on the basis of molecular patterns through either the inner or outer face of the LRR structure (Bella et al. 2008; Ng and Xavier 2011). Our molecular modeling supported that the LRR domain of mega-TSP can adopt the characteristic curved structure with distinct concave and convex faces. Molecular models of the C-terminal regions of two mega-TSPs and a sushi-TSP predicted that these polypeptide sequences would form structures highly related to the equivalent regions of canonical TSPs. For TSP1, RGD-dependent cell attachment and spreading depends on extracellular calcium ion concentration and the status of intramolecular disulfide bonds (Sun et al. 1992; Kvansakul et al. 2004). The models from mega-TSPs predicted that the equivalent loop is longer and does not provide the charge environment to support coordination of a calcium ion. A RGD motif is also lacking at the site, yet the equivalently positioned motif is VGD, which has been related to β1 integrin binding in the integrin-inhibitory activities of certain disintegrins from snakes or blood-sucking insects (Calvete et al. 2003; Assumpcao et al. 2012). The KGD motif located in the penultimate type 3 repeat of certain TSPs also coordinates a calcium ion (Kvansakul et al. 2004; Carlson et al. 2005). The mega-TSP models predict that the environment of this motif would support calcium ion binding, yet the equivalent motif is IGD. This motif also has potential to bind integrins, as IGD motifs within the type I domains of fibronectin support fibroblast migration involving αvβ3 integrin (Millard et al. 2007; Maurer et al. 2012). Thus, whereas specific aspects of Ca-coordination appear distinct, integrin binding appears a likely conserved property.

In view that mega-TSP has a much wider phylogenetic range than poriferan-TSP and sushi-TSP, mega-TSP was chosen for molecular and biological studies. We focused on cnidarians because of their value to study fundamental aspects of tissue organization. Expression patterns of *mega-TSP* transcripts were examined in *N. vectensis* and *H. magnipapillata*, each of which has distinct advantages for study of developmental stages (*Nematostella*) or regeneration of adult polyps (*Hydra*). In situ hybridization demonstrated widespread and dynamically regulated expression of *NvMT* during development, with transcripts confined to the endoderm until late stage polyps, with graded expression increasing toward the distal ends of tentacles. Ectodermal expression was prominent in the mesenteries of adult *N. vectensis* polyps, and ectodermal expression was conserved in the body wall of *Hydra* polyps. Distinctions in the patterns might reflect subfunctionalization of NvMT due to the greater number of TSP superfamily members expressed by *N. vectensis* (Tucker et al. 2013). The ectodermal expression of *HmMT* along the entire longitudinal axis of the *Hydra* is reminiscent of the expression patterns of major fibrillar collagens, *Hcol1*, *Hcol2*, and *Hcol3* (Deutzmann et al. 2000; Zhang et al. 2007; Tucker and Adams 2014), indicating that HmMT might form part of the interstitial matrix of the mesoglea, rather than the basal lamina which is produced from the endoderm (Sarras et al. 1994). In addition, *HmMT* transcript, similar to other ECM-encoding transcripts, was identified to be increased during head regeneration.

We focused on hydra to examine the function of *HmMT*. In cnidarian polyps, a single oral–aboral body axis controls patterning of the head and foot regions. This axis is set up by a constant, positive feedback loop of Wnt3 production at the hypostome as the major driver of head organization, in conjunction with a gradient of foot inhibitor from the base of the polyp (Bode 2011). Upon transection of the body column in the gastric region, gradient repatterning at the transection site activates dynamic restructuring of mesoglea and cell layers to generate a new head on the basal body fragment and a new foot on the head fragment (Sarras et al. 1991; Aufschnaiter et al. 2011). Upon siRNA-mediated silencing of *HmMT* in adult polyps, a striking necessity for *HmMT* to support head regeneration was discovered. Identical phenotypes were obtained with three pairwise combinations of siRNA to *HmMT*, supporting that these resulted from on-target effects. The need to stimulate tissue remodeling to reveal an acute phenotype has been documented for other hydra transcripts with functions in axis determination and may relate to the native rate of protein turnover in the head region (Aufschnaiter et al. 2011). Dysfunctional stem cell migration could play a role in the observed effect of *HmMT* silencing. Zhang and Sarras (1994) showed that perturbation of ECM structure in hydra, with RGDS synthetic peptide or an antibody to fibronectin, dramatically inhibited the migration of interstitial stem cells (i cells). Although hydras depleted of i cells can regenerate, normal head regeneration involves i cell activity (Chera et al. 2009). Alternatively, as head regeneration is dependent on de novo synthesis of mesoglea, a disordered basement membrane or interstitial matrix assembly caused by the lack of HmMT could block morphollaxis of the epithelial cells, which is a key process in the early phase of head regeneration (Agata et al. 2007).

Considering together the domain architectures and motifs, molecular modeling predictions, molecular phylogenetic data and analysis of transcribed sequences, models were formulated for the evolution of the TSP superfamily. With regard to the “ctenophore-sister” model of animal phylogeny (Whelan et al. 2017) and that mega-TSP was the sole form identified in ctenophores, one model is that a mega-TSP-like encoding sequence originated in the last metazoan common ancestor and that the TSP superfamily then evolved by multiple gene duplication events and subsequent domain shuffling that led to the evolution of paralogs with distinct domain compositions (fig. 7*C*). In this model, the gene duplication giving rise to sushi-TSP in modern poriferans and cnidarians occurred after the divergence of ctenophores (i.e., in the last common ancestor of poriferans, placozoans, cnidarians, and bilaterians), with subsequent loss of this gene from all lineages except poriferans and cnidarians. The absence of a coiled-coil domain from mega-TSP, sushi-TSP, and poriferan-TSP indicates that oligomerization by this mechanism is a late-evolving property specific to the TSPs.

Alternative considerations, based on the “poriferan-sister” model (Feuda et al. 2017), involve more complexity of the ancestral state, evolutionary divergences, and gene losses. Because mega-TSP and sushi-TSP are both represented in extant poriferans, an evolutionary “big bang” giving rise to mega-TSP and sushi-TSP ancestors in the metazoan common ancestor is proposed (fig. 7*D*). In this model, the mega-TSP-only state of ctenophores would result from gene loss. Assuming poriferan-TSP arose from a poriferan-specific gene duplication (discussed further below), the origin of canonical TSPs in cnidarians is posited to have resulted from gene duplication and rapid domain losses/domain shuffling and divergence of sushi-TSP. However, the molecular phylogenetic data do not clearly support this proposal.

The models also consider the conservation or absence of the DDD calcium-binding motif of the TSP L-lectin/Con-A like domain. This motif was absent from mega-TSPs. Because a DDD motif is apparent in the single Con A-like domain of poriferan-TSP and is variably present in sushi-TSP, we propose that this motif debuted in a common ancestor of sushi-TSP, and the poriferan-TSP/TSP ancestor, with subsequent lineage-specific losses (fig. 7*C*). Gene duplication and domain shuffling/loss is proposed to have given rise to sushi-TSP (with two Con A-like domains) and the ancestor of poriferan-TSP and TSPs (with a single Con A-like domain). Duplication of the latter gene in the last common ancestor of poriferans, cnidarians, and bilaterians is proposed to have given rise to poriferan-TSP and canonical TSP, with loss of the poriferan-TSP-encoding gene from the cnidarian/bilaterian common ancestor (fig. 7*C*). In the “poriferan-first” model, the same considerations lead to the proposal that TSPs arose from a duplication of the sushi-TSP gene (fig. 7*D*).

With regard to the evolution of poriferan-TSP, our analysis demonstrated that all three categories of TSP-related proteins are expressed in this phylum. As others have noted more generally, there was very high in-clade sequence variation between different classes of Porifera (Renard et al. 2018). Different poriferan species encode distinct profiles of TSP-related proteins and yet lack canonical TSPs. In the above models, we take the parsimonious assumption that canonical TSPs debuted in cnidarians. However, the presence of TSR domains in poriferan-TSPs, and also in subgroup A TSPs of deuterostomes, poses an interesting issue of whether poriferan-TSPs represent orthologs or paralogs of subgroup A TSPs. A conceivable alternative would be that poriferan-TSP also arose in the metazoan common ancestor, with subsequent gene loss in ctenophores and a convoluted evolutionary pathway involving loss of TSR domains and the origin of a B-like TSP in the cnidarian/bilaterian common ancestor. The prior model for TSP evolution is that subgroup A TSPs arose in the last deuterostome common ancestor (Bentley and Adams 2010) and several lines of evidence support this view. Conservation of synteny is apparent for each of the five TSP genes of vertebrates, and the concept that these genes arose by duplications early in the vertebrate lineage is supported by the paralogous gene locations of *THBS1*, *THBS3*, *THBS4*, and *COMP/THBS5* within the human genome (McKenzie et al. 2006). The present molecular phylogenetic analysis indicates that poriferan-TSPs form a separate clade to the canonical TSPs, whereas subgroup A TSPs clearly place closely to the pentameric TSPs (fig. 7*C*). Thus, it seems most plausible that poriferan-TSP evolved separately in the sponge ancestor and represents a phylum-specific paralog. Overall, the data reveal for the first time that TSPs represent a discrete branch within a superfamily of previously unappreciated complexity and diversity, which appears to have originated at the debut of the Metazoa. The functional significance of *Hydra* mega-TSP implicates major roles in tissue patterning or dynamics. Further study of this superfamily may illuminate principles of metazoan tissue organization.

## Materials and Methods

### Identification of TSP-Related Proteins by Bioinformatics

BlastP or TBlastN searches were conducted with the C-terminal regions (comprising the last EGF domain to the C-terminus), of *N. vectensis* Nv85341 (XP_1639928) (Tucker et al. 2013), *H. vulgaris* TSP (XP_002164610), also human TSP1 (NP_003237.2), human TSP5 (NP_000086.2), and *C. intestinalis* TSP-DD (NP_001265897.1) (Bentley and Adams 2010) as query sequences against the NCBI GenBank nucleotide, proteins or transcribed sequence assembly (TSA) databases at default parameters. We also searched for predicted TSP-related proteins in the ctenophores *Mnemiopsis leidyi* (research.nhgri.nih.gov/mnemiopsis/) (Ryan et al. 2013), *Pleurobrachia bachei* (neurobase.rc.ufl.edu/pleurobrachi; blastp against 02_P-bachei_Filtered_Gene_Models) (Moroz et al. 2014), and NCBI-TSA transcriptome of *Homophora californiensis* (Francis et al. 2015); the poriferans *Amphimedon queenslandica* (Srivastava et al. 2010), *Ephydatia muelleri*, *S. ciliatum*, *Leucosolenia complicata*, and *Oscarella carmela* (Riesgo et al. 2014) at http://www.compagen.org (Hemmrich and Bosch 2008); corals *Acropora digitifera* (Shinzato et al. 2011) and *Stylophora pistillata* (Karako-Lampert et al. 2014); *Lingula anatina*, *Phoronis australis*, and *Notospermus* geniculatus (Luo et al. 2018); acorn worm *Ptychodera flava* (Simakov et al. 2015) and Japanese pearl oyster *Pinctada fucata* (Takeuchi et al. 2012) at marinegenomics.oist.jp/genomes (Koyanagi et al. 2013); Japanese lamprey *Lethenteron japonicum* at jlampreygenome.imcb.a-star.edu.sg (Mehta et al. 2013), and choanoflagellates *Monosiga brevicollis* (Ryan et al. 2013) and *Salpingoaecea rosetta* (Fairclough et al. 2013), and the filasterean *Capsaspora owczarzaki* (Suga et al. 2013) at NCBI. In addition to canonical TSPs, these searches identified, in certain species, predicted proteins with e values of <1e-100 and sequence identities around 40–46% against human TSPs (coverage of TSPs, around 22% for mega-TSPs, 42% for sushi-TSPs, and 60% to 80% for poriferan-TSPs). These proteins were identified by InterProScan 5 (Jones et al. 2014) (ebi.ac.uk) to contain additional domains unrelated to those of TSPs. Further systematic reciprocal BlastP searches of GenBank, the databases listed above and TBlastN searches of NCBI dbest and TSA were carried out with each of the newly identified sequences to confirm relatedness to TSPs, transcription of the predicted gene products, and to garner identification of additional related proteins. The phylogenetic range of the predicted TSP-related proteins is represented in table 1. The nucleotide sequence of *HmMT* transcript was verified by searching the *H. magnipapillata* strain 105 transcriptome (NCBI BioProject PRJNA213706) with the genome-predicted protein sequence, XP_012565470. The GenBank TSA is GAOL01026196.1 (*H. magnipapillata* Seq37942.0 transcribed RNA sequence). The domain composition of all validated protein hits was ascertained via CDD (Marchler-Bauer et al. 2017) and InterProScan 5. Predicted secretory signal peptides were confirmed through the Centre for Biological Sequence analysis prediction services (http://www.cbs.dtu.dk/services/) (Petersen et al. 2011). Inspection for coiled-coil domains was carried out in MARCOIL (Delorenzi and Speed 2002).

### Multiple Sequence Alignment and Phylogenetic Trees

Data sets included the TSPs listed above and TSP-related proteins from the set in table 1 that represented the phylogenetic range of the predicted proteins. Multiple sequence alignments of amino acid sequence regions including the last EGF domain, the TSP type 3 repeats and the first L-type lectin-like/Con A domain, or of single Con A-like domains, were prepared in MUSCLE 3.8 (Edgar 2004) or webPRANK (Löytynoja and Goldman 2010) at default parameters through the resources of EBI. For preparation of phylogenetic trees, variations present in <5% of the sequences leading to gapping were removed. Phylogenetic trees were constructed by the maximum-likelihood method in PhyML 3.0 (Guindon et al. 2010) at default parameters with 200 cycles of boot-strapping. Tree diagrams were rendered from the Newick outputs in iTOL (Letunic and Bork 2011).

### Molecular Modeling

The extended N-terminal region spanning residues 108–971 of HmMT, contained predicted LRRs that guided the modeling of this region. HHPRED and Modeler (under the Max Planck Institute Bioinformatics Toolkit, https://toolkit.tuebingen.mpg.de/#/) were used to provide model segments based on the following: HmMT residues 108–359 (SDAIT to LTELW), modeled on pdb 4V2D (Seiradake et al. 2014) with 29% sequence identity; the region 360–528 (LGSN to STID), on pdb 2O6Q (Kim et al. 2007) with 24% identity; the region 529–691 (LRSNS to GPNSE), on pdb 4MN8 (Sun et al. 2013) with 27% identity; the region 692–837 (EILH to AEMS), on 4FDW (the crystal structure of a putative cell surface protein (Bacova_01565) from Bacteroides Ovatus Atcc 8483 at 2.05 A resolution), with 25% identity, and the region 838–1,016 on pdb 4MN8, with 27% identity. The segments were then conjoined by hand, guided by the repeating fold. The resulting model was energy minimized in water and 150-mM NaCl in Gromacs 4.6.7 (van der Spoel, E. Lindahl, B. Hess, and the GROMACS development team, www.gromacs.org) (Hess et al. 2008), with reasonable energies, indicating that there were no clashes or unreasonable bonds. The modeling of C-terminal regions focused in each case on the sequence from the start of the type 3 repeats to the C-terminus of the second Con A-like domain. The models for HmMT, NvMT, and ScST (scpid30246) C-terminal domain sequences were produced using HHPRED sequence alignment software (Söding et al. 2005) and Modeler (Webb and Sali 2014) (https://salilab.org/modeller/). The HmMT model shared 42% sequence identity with pdb 3FBY, the C-terminal region of human cartilage oligomeric matrix protein, also known as thrombospondin-5 (TSP-5) (Tan et al. 2009), between residues DNCEY and LQVR (designated as the TSP-like C-terminal domain). The second ConA-like domain of HmMT (from residues CLERMN to FLSQK) was modeled from pdb 4DQA, with which the HmMT second Con A domain shared 20.7% sequence identity. The C-terminal region from NvMT shared 46% identity with pdb 3FBY and was modeled between residues DNCP to EAKCA on this structure. The putative second ConA-like domain of NvMT was modeled for the region from residues VNQAL to FFVTYP against pdb 4DQA, with which it shared 21% identity. The first Con A-like domain from ScST (scpid30246; ScST) was modeled from residues SSSSVEC to CLRN on 3FBY, with which it shared 49% sequence identity. The second ConA-like domain of ScST, from TVDL to GPPRT, shared only 18% identity with the nearest structure, 4DQA, which was considered too low to produce a meaningful model.

All the resulting models were read into the Sybyl suite of software (Sybylx2.1.1, Tripos Inc.) where they were inspected. Cysteine residues likely to form disulfides were oriented within optimal reach of each other, oxidized, and disulfide bonds formed. The models were then minimised and overlaid for comparison with two TSP structures from pdb, neither of which had been used in model building: 1UX6 (TSP1 C-terminal fragment; Kvansakul et al. 2004) and 1YO8 (TSP2 C-terminal region; Carlson et al. 2005). The Hm model was further supported by the superposition of the DNC repeats with the native PDB 3FBY and 1YO8 crystal structures (1YO8 was not used to produce the model) in which overlying (conserved) disulfides constrain the structure and may stabilize potential calcium-binding sites. Models were visualized and figures prepared with the UCSF Chimera package (Pettersen et al. 2004) (http://www.cgl.ucsf.edu/chimera). Procheck (Laskowski et al. 1993) was run from the CCP4 suite of software (http://www.ccp4.ac.uk/dist/html/procheck_man/index.html).

### Laboratory Culture of Cnidarians and siRNA Treatment

#### Hydra *Species*

*Hydra magnipapillata* strain 105 and transgenic *H. vulgaris* AEP strain were cultured in hydra medium (HM) (1 mM CaCl_2_, 0.1 mM MgCl_2_, 0.1 mM KCl, 1 mM NaH_2_CO_3_, pH 7.8) at 18 °C and fed two to three times a week with freshly hatched *Artemia salina* nauplii. Before experiments, animals were starved for 24 h. For qPCR, RNA was prepared as described in Lommel et al. (2018). For siRNA experiments, a transgenic *H. vulgaris* strain expressing ectodermal GFP (actin::GFP) and endodermal RFP (actin::RFP) (Carter et al. 2016), provided by Robert Steele’s laboratory was used. siRNAs were designed for the HmMT C-terminal region (siHmMT1), the LRR domain region (siHmMT2) and the region between the LRR and EFG domains (siHmMT3). siRNAs for GFP and HmMT were purchased from Sigma-Aldrich/Merck at HPLC grade (see supplementary table S2, Supplementary Material online, for all siRNA sequences). Electroporation was performed as described (Lommel et al. 2018). 3 μM siRNA in total (1 μM si*GFP* and 2 μM scrambled si*GFP* or combinations of 1 μM each of si*HmMT*1, si*HmMT*2, and si*HmMT*3) was added to the cuvette. Twenty four hours after electroporation, living animals (*n* = 20) were transferred into a new Petri dish containing hydra medium and kept under normal hydra conditions. The feeding routine was restarted 2 days after electroporation. For the regeneration experiments, animals were allowed to recover for 7 days and fed at least four times. The animals were cut at 50% body length using a sterile scalpel blade (NeoLab sterile surgical blade No. 15). After three washing steps in hydra medium for 10 min at 18 °C, the head and foot regenerates, respectively, were kept separately in Petri dishes containing hydra medium. Regeneration was documented after 72 and 96 h, using a Nikon SMZ25 stereomicroscope equipped with a Nikon DS-Ri2 high-definition camera.

#### Nematostella vectensis

A colony of mature *N. vectensis* polyps descended from clones CH2 and CH6 from the University of California Davis Bodega Bay Marine Laboratory were raised as described (Hand and Uhlinger 1992). *Nematostella* polyps and embryos in the Heidelberg laboratory were kept in 1/3 seawater (Tropic Marine) at 18 °C in the dark and fed once or twice a week with freshly hatched *Artemia* nauplii.

### Molecular Cloning of NvMT

Cloning and sequencing of a partial cDNA for NvMT was reported in Tucker et al. (2013). The 499-bp sequence corresponds to a region of the TSP type 3 repeats. A potentially complete cDNA sequence for NvMT extending 5′ and 3′ from the 499-bp sequence was determined using DNA oligonucleotide primer pairs based on a predicted protein (JGI NemV1 198683) as well as by manual inspection of the surrounding *N. vectensis* genomic sequence. The oligonucleotide primer pairs (synthesized by Eurofins) used were 5′-aacttttaaaacacggcatgg-3′ and 5′-ggcagttgatcaaagggtgt-3′; 5′-ttgcgccacaacaagctat-3′ and 5′-tcgaatgtaggacagttggttc-3′; 5′-tcaagaataatcgcctgacca-3′ and 5′-aaaagcatcttggccgatgt-3′; 5′-acgccagggacctagatttt-3′ and 5′-gattgcgtctgtgtacccct-3′; 5′-tctacagtggccccacctac-3′ and 5′-gtcgcctcatagtccagctc-3′; 5′-ccacgatggctacatcacac-3′ and 5′-atctggacagctctcgcatt-3′; 5′-gtgcttcccaggagtgaagt-3′ and 5′-caccatcgctatcagtgtcg-3′; 5′-gtgcttgcgacactgatagc-3′ and 5′-tcctttgatcccaacattga-3′; and 5′-ggagaaaagaaaacatcaatttcg-3′ and 5′-cgtgtacggtctataaatctctgg-3′. The polyA+ RNA template for reverse transcription/polymerase chain reaction (RT-PCR) was made from 2-month-old juvenile *N. vectensis* polyps with an RNeasy Mini Kit (Qiagen) and was treated with DNase I (Life Technologies) before use. RT-PCR was carried out using a One-Step RT-PCR Kit (Qiagen) and the following amplification parameters: 1 min at 94 °C, 1 min at 50 °C, and 1.5 min at 72 °C, for 40 cycles in a MJ Mini cycler. PCR products were subcloned into pCRII with a TOPO PCR Cloning Kit (Life Technologies) and the inserts were sequenced on both strands at UC Davis Sequencing. When assembled, the nine overlapping cDNAs correspond to an 8,509-bp nucleotide sequence encoding a 2,804 aa ORF. The full sequence is deposited as GenBank accession MF962901.

### In Situ Hybridization

#### Nematostella vectensis

Adults: Sexually mature *N. vectensis* were raised and prepared for in situ hybridization as described (Tucker and Gong 2014). The fixed tissues were infiltrated and embedded in Paraplast (Fisher Scientific) and 8-μm-thick sections collected on Superfrost Plus (Fisher Scientific) glass slides. Sections were treated with Proteinase K at room temperature for 3 min. After postfixation, rinsing and dehydration, 1 μg/ml of riboprobe (from the pCRII plasmid containing the NvMT nucleic acid region encoding aa 956–1,275 of NvMT, which is unique to mega-TSPs to minimize the possibility of cross-hybridization with canonical TSP) was added to the slides and hybridization carried out at 65 °C overnight followed by colorization. Larvae and primary polyps: In situ hybridization of planula larvae and primary polyps of *N. vectensis* was performed as described (Watanabe et al. 2014). In brief, specimens were fixed with 4% paraformaldehyde/PBST (PBS, 0.1% Tween 20) for 1 h at room temperature. Hybridizations were carried out in a hybridization solution containing 1% SDS at 60 °C for at least 22 h. Sense (control) and antisense digoxigenin-labeled RNA probes corresponded to the region encoding aa 956–1,275 of NvMT.

#### Hydra magnipapillata

Customized LNA digoxygenin-labeled RNA probes were designed and produced by Exiqon (5′DigN/AGTAAACTTGTGCTGTAGATGT/3′Dig_N; Position in target: 1,207–1,229) corresponding to the antisense strand of *HmMT* cDNA. The whole-mount in situ hybridization procedure was performed as described previously (Schüler et al. 2015). For hybridization, the LNA probe was added to a final concentration of ∼1 µM in fresh HS and hybridized for ∼60 h at 55 °C.

### Vibratome Sectioning of Hydra

Freshly in situ-stained *Hydra* polyps were embedded in 1.5 ml PBS containing 0.5% gelatin, 30% bovine albumin-fraction V (Carl Roth) and solidified with 105 μl glutaraldehyde (Sigma). Cross sections (100 μm) were cut from the midgastric region with a VT1000 S vibrating blade microtome (Leica) and mounted on microscope slides in Mowiol 4-88(Carl Roth).

### Microscopy and Image Processing

Images were captured with a Nikon Eclipse 80i or E800 photomicroscope using NIC filters and a Nikon Digital Sight DS-U1 color camera and processed with Adobe Photoshop CS6 and ImageJ.

## Supplementary Material

Supplementary data are available at *Molecular Biology and Evolution* online.

## Supplementary Material

Supplementary_Material_msz060

## References

[msz060-B1] Adams JC, Lawler J. 2011 The thrombospondins. Cold Spring Harb Perspect Biol. 3:a009712. 21875984 10.1101/cshperspect.a009712 PMC3179333

[msz060-B2] Agata K, Saito Y, Nakajima E. 2007 Unifying principles of regeneration I: epimorphosis versus morphallaxis. Dev Growth Differ. 49 2: 73–78. 17335428 10.1111/j.1440-169X.2007.00919.x

[msz060-B3] Assumpcao TC, Ribeiro JM, Francischetti IM. 2012 Disintegrins from hematophagous sources. Toxins (Basel) 4 5: 296–322. 22778902 10.3390/toxins4050296 PMC3386632

[msz060-B4] Aufschnaiter R, Zamir EA, Little CD, Özbek S, Münder S, David CN, Li L, Sarras MP Jr, Zhang X. 2011 In vivo imaging of basement membrane movement: ECM patterning shapes *Hydra* polyps. J Cell Sci. 124(Pt 23): 4027–4038. 22194305 10.1242/jcs.087239 PMC3244984

[msz060-B5] Beckmann G, Bork P. 1993 An adhesive domain detected in functionally diverse receptors. Trends Biochem Sci. 18 2: 40–41. 8387703 10.1016/0968-0004(93)90049-s

[msz060-B6] Bella J, Hindle KL, McEwan PA, Lovell SC. 2008 The leucine-rich repeat structure. Cell Mol Life Sci. 65 15: 2307–2333. 18408889 10.1007/s00018-008-8019-0 PMC11131621

[msz060-B7] Bentley AA, Adams JC. 2010 The evolution of thrombospondins and their ligand-binding activities. Mol Biol Evol. 27 9: 2187–2197. 20427418 10.1093/molbev/msq107 PMC3107593

[msz060-B8] Bertrand S, Fuentealba J, Aze A, Hudson C, Yasuo H, Torrejon M, Escriva H, Marcellini S. 2013 A dynamic history of gene duplications and losses characterizes the evolution of the SPARC family in eumetazoans. Proc Biol Sci. 280:2012.2963. 10.1098/rspb.2012.2963 PMC3619480 23446527

[msz060-B9] Bode H. 2011 Axis formation in hydra. Annu Rev Genet. 45:105–117. 21819240 10.1146/annurev-genet-102209-163540

[msz060-B10] Brunet T, King N. 2017 The origin of animal multicellularity and cell differentiation. Dev Cell 43 2: 124–140. 29065305 10.1016/j.devcel.2017.09.016 PMC6089241

[msz060-B11] Calvete JJ, Moreno-Murciano MP, Theakston RD, Kisiel DG, Marcinkiewicz C. 2003 Snake venom disintegrins: novel dimeric disintegrins and structural diversification by disulphide bond engineering. Biochem J. 372(Pt 3): 725–734. 12667142 10.1042/BJ20021739 PMC1223455

[msz060-B12] Carlson CB, Bernstein DA, Annis DS, Misenheimer TM, Hannah BL, Mosher DF, Keck JL. 2005 Structure of the calcium-rich signature domain of human thrombospondin-2. Nat Struct Mol Biol. 12 10: 910–914. 16186819 10.1038/nsmb997 PMC2219892

[msz060-B13] Carter JA, Hyland C, Steele RE, Collins EM. 2016 Dynamics of mouth opening in *Hydra*. Biophys J. 110 5: 1191–1201. 26958895 10.1016/j.bpj.2016.01.008 PMC4788721

[msz060-B14] Chanana B, Graf R, Koledachkina T, Pflanz R, Vorbrüggen G. 2007 AlphaPS2 integrin-mediated muscle attachment in *Drosophila* requires the ECM protein Thrombospondin. Mech Dev. 124 6: 463–475. 17482800 10.1016/j.mod.2007.03.005

[msz060-B15] Chapman JA, Kirkness EF, Simakov O, Hampson SE, Mitros T, Weinmaier T, Rattei T, Balasubramanian PG, Borman J, Busam D. 2010 The dynamic genome of *Hydra*. Nature 464 7288: 592–596. 20228792 10.1038/nature08830 PMC4479502

[msz060-B16] Chera S, Ghila L, Dobretz K, Wenger Y, Bauer C, Buzgariu W, Martinou JC, Galliot B. 2009 Apoptotic cells provide an unexpected source of Wnt3 signaling to drive *Hydra* head regeneration. Dev Cell 17 2: 279–289. 19686688 10.1016/j.devcel.2009.07.014

[msz060-B17] Delorenzi M, Speed T. 2002 An HMM model for coiled-coil domains and a comparison with PSSM-based predictions. Bioinformatics 18 4: 617–625. 12016059 10.1093/bioinformatics/18.4.617

[msz060-B18] Deutzmann R, Fowler S, Zhang X, Boone K, Dexter S, Boot-Handford RP, Rachel R, Sarras MP Jr. 2000 Molecular, biochemical and functional analysis of a novel and developmentally important fibrillar collagen (Hcol-I) in hydra. Development 127 21: 4669–4680. 11023869 10.1242/dev.127.21.4669

[msz060-B19] Dohrmann M, Wörheide G. 2017 Dating early animal evolution using phylogenomic data. Sci Rep. 7 1: 3599. 28620233 10.1038/s41598-017-03791-w PMC5472626

[msz060-B20] Edgar RC. 2004 MUSCLE: multiple sequence alignment with high accuracy and high throughput. Nucleic Acids Res. 32 5: 1792–1797. 15034147 10.1093/nar/gkh340 PMC390337

[msz060-B21] Exposito JY, Valcourt U, Cluzel C, Lethias C. 2010 The fibrillar collagen family. Int J Mol Sci. 11 2: 407–426. 20386646 10.3390/ijms11020407 PMC2852846

[msz060-B22] Fairclough SR, Chen Z, Kramer E, Zeng Q, Young S, Robertson HM, Begovic E, Richter DJ, Russ C, Westbrook MJ, et al 2013 Premetazoan genome evolution and the regulation of cell differentiation in the choanoflagellate *Salpingoeca rosetta*. Genome Biol. 14 2: R15. 23419129 10.1186/gb-2013-14-2-r15 PMC4054682

[msz060-B23] Feuda R, Dohrmann M, Pett W, Philippe H, Rota-Stabelli O, Lartillot N, Wörheide G, Pisani D. 2017 Improved modeling of compositional heterogeneity supports sponges as sister to all other animals. Curr Biol. 27 24: 3864–3870.e4. 29199080 10.1016/j.cub.2017.11.008

[msz060-B24] Fidler AL, Darris CE, Chetyrkin SV, Pedchenko VK, Boudko SP, Brown KL, Gray Jerome W, Hudson JK, Rokas A, Hudson BG. 2017 Collagen IV and basement membrane at the evolutionary dawn of metazoan tissues. Elife 6:pii: e24176. 10.7554/eLife.24176 PMC5395295 28418331

[msz060-B25] Francis WR, Shaner NC, Christianson LM, Powers ML, Haddock SH. 2015 Occurrence of isopenicillin-N-synthase homologs in bioluminescent ctenophores and implications for coelenterazine biosynthesis. PLoS One 10 6: e0128742. 26125183 10.1371/journal.pone.0128742 PMC4488382

[msz060-B26] Guindon S, Dufayard JF, Lefort V, Anisimova M, Hordijk W, Gascuel O. 2010 New algorithms and methods to estimate maximum-likelihood phylogenies: assessing the performance of PhyML 3.0. Systematic Biol. 59 3: 307–321. 10.1093/sysbio/syq010 20525638

[msz060-B27] Hand C, Uhlinger KR. 1992 The culture, sexual and asexual reproduction, and growth of the sea anemone *Nematostella vectensis*. Biol Bull. 182 2: 169–176. 29303672 10.2307/1542110

[msz060-B28] Hemmrich G, Bosch T. 2008 Compagen, a comparative genomics platform for early branching metazoan animals reveals early origins of genes regulating stem cell differentiation. Bioessays 20:1010–1018. 10.1002/bies.20813 18800383

[msz060-B29] Hess B, Kutzner C, van der Spoel D, Lindahl E. 2008 GROMACS 4: algorithms for highly efficient, load-balanced, and scalable molecular simulation. J Chem Theory Comp. 4 3: 435–447. 10.1021/ct700301q 26620784

[msz060-B30] Hutchinson EG, Thornton JM. 1993 The Greek key motif: extraction, classification and analysis. Protein Eng. 6 3: 233–245. 8506258 10.1093/protein/6.3.233

[msz060-B31] Johnstone IL. 2000 Cuticle collagen genes. Expression in *Caenorhabditis elegans*. Trends Genet. 16 1: 21–27. 10637627 10.1016/s0168-9525(99)01857-0

[msz060-B32] Jones P, Binns D, Chang HY, Fraser M, Li W, McAnulla C, McWilliam H, Maslen J, Mitchell A, Nuka G, et al 2014 InterProScan 5: genome-scale protein function classification. Bioinformatics 30 9: 1236–1240. 24451626 10.1093/bioinformatics/btu031 PMC3998142

[msz060-B33] Karako-Lampert S, Zoccola D, Salmon-Divon M, Katzenellenbogen M, Tambutté S, Bertucci A, Hoegh-Guldberg O, Deleury E, Allemand D, Levy O. 2014 Transcriptome analysis of the scleractinian coral *Stylophora pistillata*. PLoS One 9 2: e88615. 24551124 10.1371/journal.pone.0088615 PMC3923803

[msz060-B34] Kim DJ, Christofidou ED, Keene DR, Milde HM, Adams JC. 2015 Inter-molecular interactions of thrombospondins drive their accumulation in extracellular matrix. Mol Biol Cell 26 14: 2640–2654. 25995382 10.1091/mbc.E14-05-0996 PMC4501361

[msz060-B35] Kim HM, Oh SC, Lim KJ, Kasamatsu J, Heo JY, Park BS, Lee H, Yoo OJ, Kasahara M, Lee JO. 2007 Structural diversity of the hagfish variable lymphocyte receptors. J Biol Chem. 282 9: 6726–6732. 17192264 10.1074/jbc.M608471200

[msz060-B36] King N, Westbrook MJ, Young SL, Kuo A, Abedin M, Chapman J, Fairclough S, Hellsten U, Isogai Y, Letunic I, et al 2008 The genome of the choanoflagellate *Monosiga brevicollis* and the origin of metazoans. Nature 451 7180: 783–788. 18273011 10.1038/nature06617 PMC2562698

[msz060-B37] Koyanagi R, Takeuchi T, Hisata K, Gyoja F, Shoguchi E, Satoh N, Kawashima T. 2013 MarinegenomicsDB: an integrated genome viewer for community-based annotation of genomes. Zool Sci. 30 10: 797–800. 24125644 10.2108/zsj.30.797

[msz060-B38] Kvansakul M, Adams JC, Hohenester E. 2004 Structure of a thrombospondin-1 C-terminal fragment reveals a novel calcium core in the type 3 repeats. EMBO J. 23 6: 1223–1233. 15014436 10.1038/sj.emboj.7600166 PMC381422

[msz060-B39] Laskowski RA, MacArthur MW, Moss DS, Thornton JM. 1993 PROCHECK: a program to check the stereochemical quality of protein structures. J Appl Cryst. 26 2: 283–291.

[msz060-B40] Lawler J, Hynes RO. 1989 An integrin receptor on normal and thrombasthenic platelets that binds thrombospondin. Blood 74 6: 2022–2027. 2478219

[msz060-B41] Layden MJ, Rentzsch F, Röttinger E. 2016 The rise of the starlet sea anemone *Nematostella vectensis* as a model system to investigate development and regeneration. Wiley Interdiscip Rev Dev Biol. 5 4: 408–428. 26894563 10.1002/wdev.222 PMC5067631

[msz060-B42] Letunic I, Bork P. 2011 Interactive Tree of Life v2: online annotation and display of phylogenetic trees made easy. Nucleic Acids Res. 39(Web Server issue): W475–W478. 21470960 10.1093/nar/gkr201 PMC3125724

[msz060-B43] Lommel M, Strompen J, Hellewell AL, Balasubramanian GP, Christofidou ED, Thomson AR, Boyle AL, Woolfson DN, Puglisi K, Hartl M, et al 2018 Hydra mesoglea proteome identifies thrombospondin as a conserved component active in head organizer restriction. Sci Rep. 8 1: 11753. 30082916 10.1038/s41598-018-30035-2 PMC6079037

[msz060-B44] Löytynoja A, Goldman N. 2010 webPRANK: a phylogeny-aware multiple sequence aligner with interactive alignment browser. BMC Bioinformatics 11:579. 21110866 10.1186/1471-2105-11-579 PMC3009689

[msz060-B45] Luo YJ, Kanda M, Koyanagi R, Hisata K, Akiyama T, Sakamoto H, Sakamoto T, Satoh N. 2018 Nemertean and phoronid genomes reveal lophotrochozoan evolution and the origin of bilaterian heads. Nat Ecol Evol. 2 1: 141–151. 29203924 10.1038/s41559-017-0389-y

[msz060-B46] MacWilliams HK. 1983 Hydra transplantation phenomena and the mechanism of Hydra head regeneration. II. Properties of the head activation. Dev Biol. 96 1: 239–257. 6825956 10.1016/0012-1606(83)90325-1

[msz060-B47] Marchler-Bauer A, Bo Y, Han L, He J, Lanczycki CJ, Lu S, Chitsaz F, Derbyshire MK, Geer RC, Gonzales NR, et al 2017 CDD/SPARCLE: functional classification of proteins via subfamily domain architectures. Nucleic Acids Res. 45(D1): D200–D203. 27899674 10.1093/nar/gkw1129 PMC5210587

[msz060-B48] Maurer LM, Annis DS, Mosher DF. 2012 IGD motifs, which are required for migration stimulatory activity of fibronectin type I modules, do not mediate binding in matrix assembly. PLoS One 7 2: e30615. 22355321 10.1371/journal.pone.0030615 PMC3280255

[msz060-B49] McKenzie P, Chadalavada SC, Bohrer J, Adams JC. 2006 Phylogenomic analysis of vertebrate thrombospondins reveals fish-specific paralogues, ancestral gene relationships and a tetrapod innovation. BMC Evol Biol. 6 1: 33. 16620379 10.1186/1471-2148-6-33 PMC1464143

[msz060-B50] Mehta TK, Ravi V, Yamasaki S, Lee AP, Lian MM, Tay BH, Tohari S, Yanai S, Tay A, Brenner S, et al 2013 Evidence for at least six Hox clusters in the Japanese lamprey (*Lethenteron japonicum*). Proc Natl Acad Sci U S A. 110 40: 16044–16049. 24043829 10.1073/pnas.1315760110 PMC3791769

[msz060-B51] Millard CJ, Ellis IR, Pickford AR, Schor AM, Schor SL, Campbell ID. 2007 The role of the fibronectin IGD motif in stimulating fibroblast migration. J Biol Chem. 282 49: 35530–35535. 17921136 10.1074/jbc.M707532200

[msz060-B52] Moroz LL, Kocot KM, Citarella MR, Dosung S, Norekian TP, Povolotskaya IS, Grigorenko AP, Dailey C, Berezikov E, Buckley KM, et al 2014 The ctenophore genome and the evolutionary origins of neural systems. Nature 510 7503: 109–114. 24847885 10.1038/nature13400 PMC4337882

[msz060-B53] Murphy-Ullrich JE, Sage EH. 2014 Revisiting the matricellular concept. Matrix Biol. 37:1–14. 25064829 10.1016/j.matbio.2014.07.005 PMC4379989

[msz060-B54] Naba A, Clauser KR, Hoersch S, Liu H, Carr SA, Hynes RO. 2012 The matrisome: in silico definition and in vivo characterization by proteomics of normal and tumor extracellular matrices. Mol Cell Proteomics 11 4: M111.014647. 10.1074/mcp.M111.014647 PMC3322572 22159717

[msz060-B55] Ng A, Xavier RJ. 2011 Leucine-rich repeat (LRR) proteins: integrators of pattern recognition and signaling in immunity. Autophagy 7 9: 1082–1084. 21606681 10.4161/auto.7.9.16464 PMC3901792

[msz060-B56] Özbek S, Balasubramanian PG, Chiquet-Ehrismann R, Tucker RP, Adams JC. 2010 The evolution of extracellular matrix. Mol Biol Cell 21 24: 4300–4306. 21160071 10.1091/mbc.E10-03-0251 PMC3002383

[msz060-B57] Petersen TN, Brunak S, von Heijne G, Nielsen H. 2011 SignalP 4.0: discriminating signal peptides from transmembrane regions. Nat Methods. 8 10: 785–786. 21959131 10.1038/nmeth.1701

[msz060-B58] Pettersen EF, Goddard TD, Huang CC, Couch GS, Greenblatt DM, Meng EC, Ferrin TE. 2004 UCSF Chimera—a visualization system for exploratory research and analysis. J Comput Chem. 25 13: 1605–1612. 15264254 10.1002/jcc.20084

[msz060-B59] Renard E, Leys SP, Wörheide G, Borchiellini C. 2018 Understanding animal evolution: the added value of sponge transcriptomics and genomics: the disconnect between gene content and body plan evolution. Bioessays 40 9: e1700237. 30070368 10.1002/bies.201700237

[msz060-B60] Riesgo A, Farrar N, Windsor PJ, Giribet G, Leys SP. 2014 The analysis of eight transcriptomes from all poriferan classes reveals surprising genetic complexity in sponges. Mol Biol Evol. 31 5: 1102–1120. 24497032 10.1093/molbev/msu057

[msz060-B61] Ryan JF, Pang K, Schnitzler CE, Nguyen A-D, Moreland RT, Simmons DK, Koch BJ, Francis WR, Havlak P, Smith SA, et al 2013 The genome of the ctenophore *Mnemiopsis leidyi* and its implications for cell type evolution. Science 342 6164: 1242592. 24337300 10.1126/science.1242592 PMC3920664

[msz060-B62] Ryu T, Seridi L, Moitinho-Silva L, Oates M, Liew YJ, Mavromatis C, Wang X, Haywood A, Lafi FF, Kupresanin M, et al 2016 Hologenome analysis of two marine sponges with different microbiomes. BMC Genomics. 17:158. 26926518 10.1186/s12864-016-2501-0 PMC4772301

[msz060-B63] Sarras MP Jr, Meador D, Zhang XM. 1991 Extracellular matrix (mesoglea) of *Hydra vulgaris*. II. Influence of collagen and proteoglycan components on head regeneration. Dev Biol. 148 2: 495–500. 1743397 10.1016/0012-1606(91)90267-7

[msz060-B64] Sarras MP Jr, Yan L, Grens A, Zhang X, Agbas A, Huff JK, St John PL, Abrahamson DR. 1994 Cloning and biological function of laminin in *Hydra vulgaris*. Dev Biol. 164 1: 312–324. 8026633 10.1006/dbio.1994.1201

[msz060-B65] Schüler A, Schmitz G, Reft A, Özbek S, Thurm U, Bornberg-Bauer E. 2015 The rise and fall of TRP-N, an ancient family of mechanogated ion channels, in Metazoa. Genome Biol Evol. 7 6: 1713–1727. 26100409 10.1093/gbe/evv091 PMC4494053

[msz060-B66] Seiradake E, del Toro D, Nagel D, Cop F, Härtl R, Ruff T, Seyit-Bremer G, Harlos K, Border EC, Acker-Palmer A, et al 2014 FLRT structure: balancing repulsion and cell adhesion in cortical and vascular development. Neuron 84 2: 370–385. 25374360 10.1016/j.neuron.2014.10.008 PMC4210639

[msz060-B67] Shinzato C, Shoguchi E, Kawashima T, Hamada M, Hisata K, Tanaka M, Fujie M, Fujiwara M, Koyanagi R, Ikuta T, et al 2011 Using the *Acropora digitifera* genome to understand coral responses to environmental change. Nature 476 7360: 320–323. 21785439 10.1038/nature10249

[msz060-B68] Simakov O, Kawashima T, Marlétaz F, Jenkins J, Koyanagi R, Mitros T, Hisata K, Bredeson J, Shoguchi E, Gyoja F, et al 2015 Hemichordate genomes and deuterostome origins. Nature 527 7579: 459–465. 26580012 10.1038/nature16150 PMC4729200

[msz060-B69] Söding J, Biegert A, Lupas AN. 2005 The HHpred interactive server for protein homology detection and structure prediction. Nucleic Acids Res. 33(Web Server issue): W244–W248. 15980461 10.1093/nar/gki408 PMC1160169

[msz060-B70] Srivastava M, Simakov O, Chapman J, Fahey B, Gauthier ME, Mitros T, Richards GS, Conaco C, Dacre M, Hellsten U, et al 2010 The *Amphimedon queenslandica* genome and the evolution of animal complexity. Nature 466 7307: 720–726. 20686567 10.1038/nature09201 PMC3130542

[msz060-B71] Stenina-Adognravi O, Plow EF. 2019. Thrombospondin-4 in tissue remodeling. *Matrix Biol*. 75-76: 300–313. 10.1016/j.matbio.2017.11.006 PMC6005712 29138119

[msz060-B72] Suga H, Chen Z, de Mendoza A, Sebé-Pedrós A, Brown MW, Kramer E, Carr M, Kerner P, Vervoort M, Sánchez-Pons N, et al 2013 The Capsaspora genome reveals a complex unicellular prehistory of animals. Nat Commun. 4:2325. 23942320 10.1038/ncomms3325 PMC3753549

[msz060-B73] Sun X, Skorstengaard K, Mosher DF. 1992 Disulfides modulate RGD-inhibitable cell adhesive activity of thrombospondin. J Cell Biol. 118 3: 693–701. 1379247 10.1083/jcb.118.3.693 PMC2289535

[msz060-B74] Sun Y, Li L, Macho AP, Han Z, Hu Z, Zipfel C, Zhou JM, Chai J. 2013 Structural basis for flg22-induced activation of the Arabidopsis FLS2-BAK1 immune complex. Science 342 6158: 624–628. 24114786 10.1126/science.1243825

[msz060-B75] Takeuchi T, Kawashima T, Koyanagi R, Gyoja F, Tanaka M, Ikuta T, Shoguchi E, Fujiwara M, Shinzato C, Hisata K, et al 2012 Draft genome of the pearl oyster *Pinctada fucata*: a platform for understanding bivalve biology. DNA Res. 19 2: 117–130. 22315334 10.1093/dnares/dss005 PMC3325083

[msz060-B76] Tan K, Duquette M, Joachimiak A, Lawler J. 2009 The crystal structure of the signature domain of cartilage oligomeric matrix protein: implications for collagen, glycosaminoglycan and integrin binding. FASEB J. 23 8: 2490–2501. 19276170 10.1096/fj.08-128090 PMC2717772

[msz060-B77] Tucker RP. 2004 The thrombospondin type 1 repeat superfamily. Int J Biochem Cell Biol. 36 6: 969–974. 15094110 10.1016/j.biocel.2003.12.011

[msz060-B79] Tucker RP, Adams JC. 2014 Adhesion networks of Cnidaria: a post-genomic view. Int Rev Cell Mol Biol. 308:323–377. 24411175 10.1016/B978-0-12-800097-7.00008-7

[msz060-B80] Tucker RP, Gong Q. 2014 Immunohistochemistry and in situ hybridization in the developing chicken brain. Methods Mol Biol. 1082:217–233. 24048937 10.1007/978-1-62703-655-9_15

[msz060-B81] Tucker RP, Hess JF, Gong Q, Garvey K, Shibata B, Adams JC. 2013 A thrombospondin in the anthozoan *Nematostella vectensis* is associated with the nervous system and upregulated during regeneration. Biol Open 2 2: 217–226. 23430283 10.1242/bio.20123103 PMC3575656

[msz060-B82] Vincent TL, Woolfson DN, Adams JC. 2013 Prediction and analysis of higher-order coiled-coils: insights from proteins of the extracellular matrix, tenascins and thrombospondins. Intl J Biochem Cell Biol. 45 11: 2392–2401. 10.1016/j.biocel.2013.07.011 23891848

[msz060-B83] Warner JF, Guerlais V, Amiel AR, Johnston H, Nedoncelle K, Röttinger E. 2018 NvERTx: a gene expression database to compare embryogenesis and regeneration in the sea anemone *Nematostella vectensis*. Development 145 10: pii: dev162867. 10.1242/dev.162867 29739837

[msz060-B84] Watanabe H, Kuhn A, Fushiki M, Agata K, Özbek S, Fujisawa T, Holstein TW. 2014 Sequential actions of β-catenin and Bmp pattern the oral nerve net in *Nematostella vectensis*. Nat Commun. 5:5536. 25534229 10.1038/ncomms6536 PMC4284808

[msz060-B85] Webb B, Sali A. 2014 Comparative protein structure modeling using modeller. Curr Protoc Bioinformatics 54:5.6.1–5.6.32. 10.1002/0471250953.bi0506s47 25199792

[msz060-B86] Whelan NV, Kocot KM, Moroz TP, Mukherjee K, Williams P, Paulay G, Moroz LL, Halanych KM. 2017 Ctenophore relationships and their placement as the sister group to all other animals. Nat Ecol Evol. 1 11: 1737–1746. 28993654 10.1038/s41559-017-0331-3 PMC5664179

[msz060-B87] Williams A, Westhead DR. 2002 Sequence relationships in the legume lectin fold and other jelly rolls. Protein Eng. 15 10: 771–774. 12468709 10.1093/protein/15.10.771

[msz060-B88] Williams F, Tew HA, Paul CE, Adams JC. 2014 The predicted secretomes of *Monosiga brevicollis* and *Capsaspora owczarzaki*, close unicellular relatives of metazoans, reveal new insights into the evolution of the metazoan extracellular matrix. Matrix Biol. 37:60–68. 24561726 10.1016/j.matbio.2014.02.002

[msz060-B89] Wouters MA, Rigoutsos I, Chu CK, Feng LL, Sparrow DB, Dunwoodie SL. 2005 Evolution of distinct EGF domains with specific functions. Protein Sci. 14 4: 1091–1103. 15772310 10.1110/ps.041207005 PMC2253431

[msz060-B90] Zhang X, Boot-Handford RP, Huxley-Jones J, Forse LN, Mould AP, Robertson DL, Lili, Athiyal M, Sarras MP Jr. 2007 The collagens of hydra provide insight into the evolution of metazoan extracellular matrices. J Biol Chem. 282:6792–6802. 17204477 10.1074/jbc.M607528200

[msz060-B91] Zhang X, Sarras MP Jr. 1994 Cell-extracellular matrix interactions under *in vivo* conditions during interstitial cell migration in *Hydra vulgaris*. Development 120 2: 425–432. 8149918 10.1242/dev.120.2.425

