# Supplementary material for: Emergence of a Thrombospondin Superfamily at the Origin of Metazoans
Source: Mol Biol Evol. 2019 Mar 13;36(6):1220–38. doi: 10.1093/molbev/msz060 (PMC6526912; doi:10.1093/molbev/msz060)
Supplement: Supplementary_Material_msz060 [file supplementary_material_msz060.zip › Supplementary Table 1.pdf]

Supplementary Table 1.
A. siRNA sequences used in this study.

**siRNA**	**Nucleotide sequence (5’ to 3’)**
si*GFP*	TAGATGGTGATGTTAATGGGC
si*HmMT*-1	AACGCTCTGTGGTACTCAGGA
si*HmMT*-2	AACGCTCTTGAAACTAGAACC
si*HmMT*-3	AATTGACTTACGCAGTAATAG

B. qPCR oligonucleotide primers used In this study.

**Transcript target**	**Nucleotide Sequence (5’ to 3’)**
HmMT fw	GCAGACGAGGTTAAAGAAGCA
HmMT rev	GCGTAGTCAATAGTACTCTCAACC
gamma-Tubulin fw	CTGCTTGTGTAGCATACTTTGAAAT
gamma-Tubulin rev	TCATGTTCAGCTACAAGAAATTCAC
