# Supplementary material for: Emergence of a Thrombospondin Superfamily at the Origin of Metazoans
Source: Mol Biol Evol. 2019 Mar 13;36(6):1220–38. doi: 10.1093/molbev/msz060 (PMC6526912; doi:10.1093/molbev/msz060)
Supplement: Supplementary_Material_msz060 [file supplementary_material_msz060.zip › SupplementaryFig1.pdf]

A

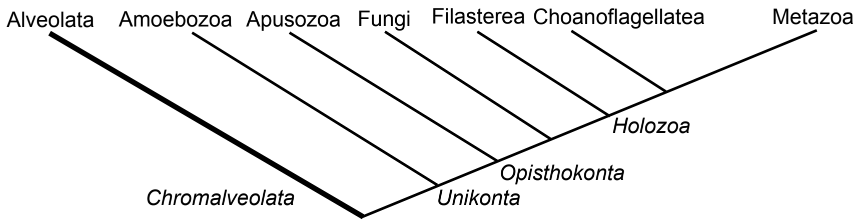

B

Domain	InterPro Code	Representation in Unikonta and Alveolata as Outgroup (InterPro release 71.0)
LN-G	IPR001791	Alveolata, Amoebozoa, Opisthokonta
TSP(N)	IPR037349	Opisthokonta (Metazoa only)
vWF_C	IPR001007	Alveolata (1 only), Opisthokonta (Metazoa only)
TSR	IPR000884	Alveolata, Amoebozoa (6 only), Apusozoa (1 only), Opisthokonta
EGF-like	IPR000742	Alveolata, Amoebozoa, Apusozoa, Opisthokonta
TSP Type 3	IPR017897	Opisthokonta (Metazoa only)
TSP-C	IPR008859	Opisthokonta (Metazoa only)
Con_A	IPR013320	Alveolata, Amoebozoa, Apusozoa, Opisthokonta
Sushi	IPR000436	Alveolata, Apusozoa, Opisthokonta
LRR	IPR001611	Alveolata, Amoebozoa, Apusozoa, Opisthokonta
Discoidin	IPR000421	Alveolata, Amoebozoa, Apusozoa (2 only), Opisthokonta
IgG/CAD-like	IPR015919	Alveolata, Amoebozoa, Apusozoa, Opisthokonta

C

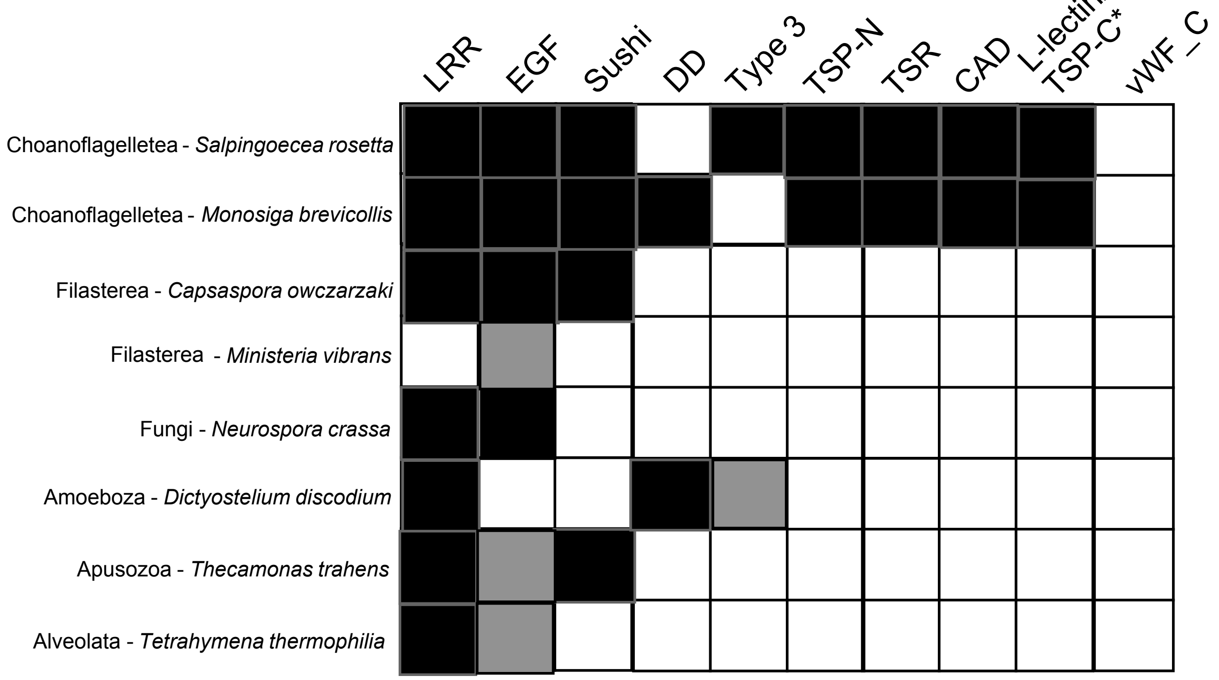

Supplementary Fig. 1. Representation of Component Domains of TSP Superfamily Members outside the Metazoa.
A, Schematic representation of the phylogenetic relationships of the closest eukaryotic relatives of the Metazoa. Chromalveolates are indicated as an outgroup to the unikonts.
B, Representation in InterPro of the domains of TSP superfamily members in the indicated eukaryotic groups.
C, Summary of results of BLASTP searches with the domains from TSP superfamily members against the indicated species of extant protists and fungi. Black squares indicate identification withh e value <0.01, grey squares indicate identification≥ 0.05.
