# Supplementary material for: Emergence of a Thrombospondin Superfamily at the Origin of Metazoans
Source: Mol Biol Evol. 2019 Mar 13;36(6):1220–38. doi: 10.1093/molbev/msz060 (PMC6526912; doi:10.1093/molbev/msz060)

Supplementary Fig. 2. MUSCLE multiple sequence alignments of conserved motifs in TSP superfamily members. Each panel displays the respective region from an alignment of the full TSP-like C-terminal region, prepared in MUSCLE 3.8 and displayed in Boxshade. Residues identical in more than 50% of the sequences have black background, conservative substitutions have grey background and non-conserved residues have white background.

A. Conservation of a KGD motif in TSP type 3 repeat 6 across members of the TSP superfamily. Protein name codes are as in Table 1.

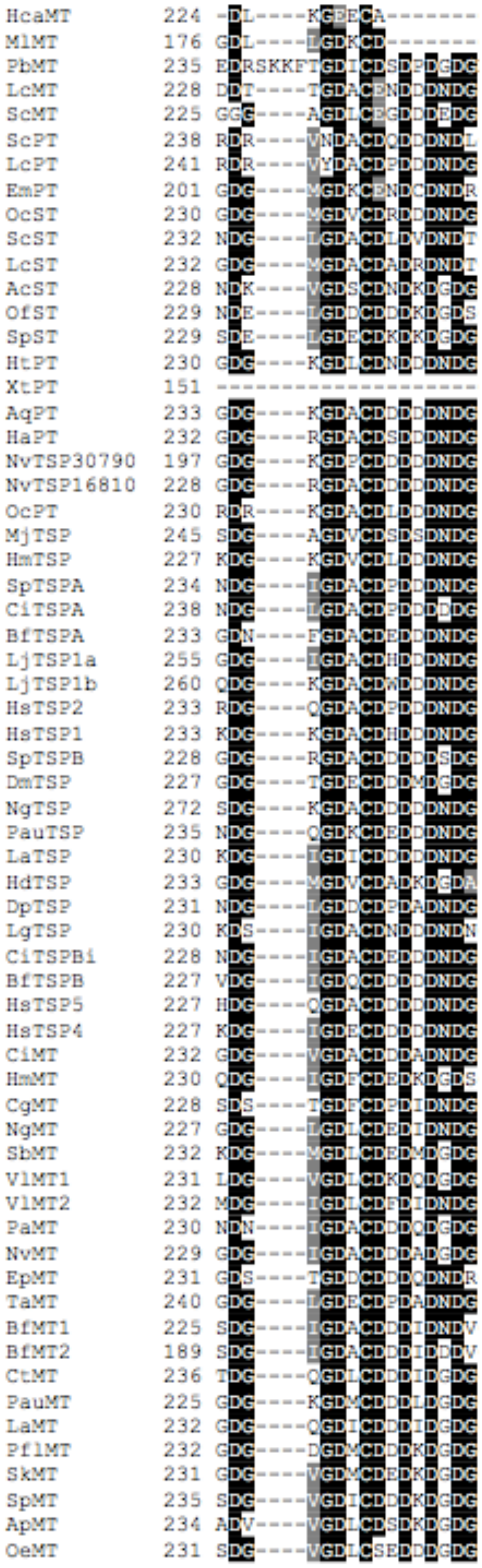

B. Conservation of an RGD motif in TSP type 3 repeat 7 across members of the TSP superfamily. Protein name codes are as in Table 1.

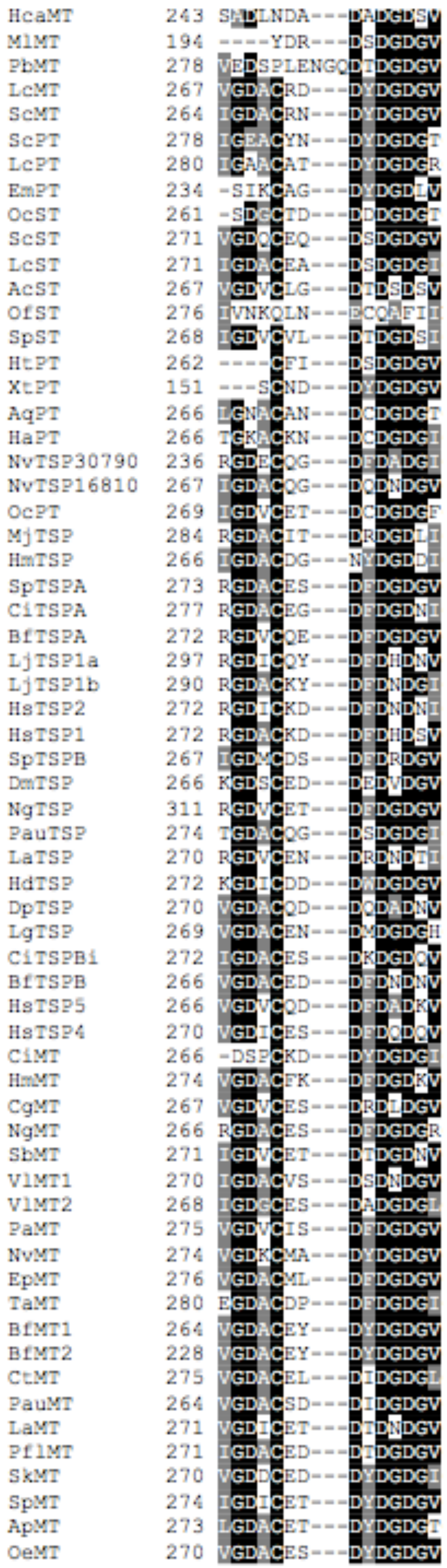

C. Conservation of the DDD motif within the first L-lectin/ConA-like domain of members of the TSP superfamily. Protein name codes are as in Table 1.

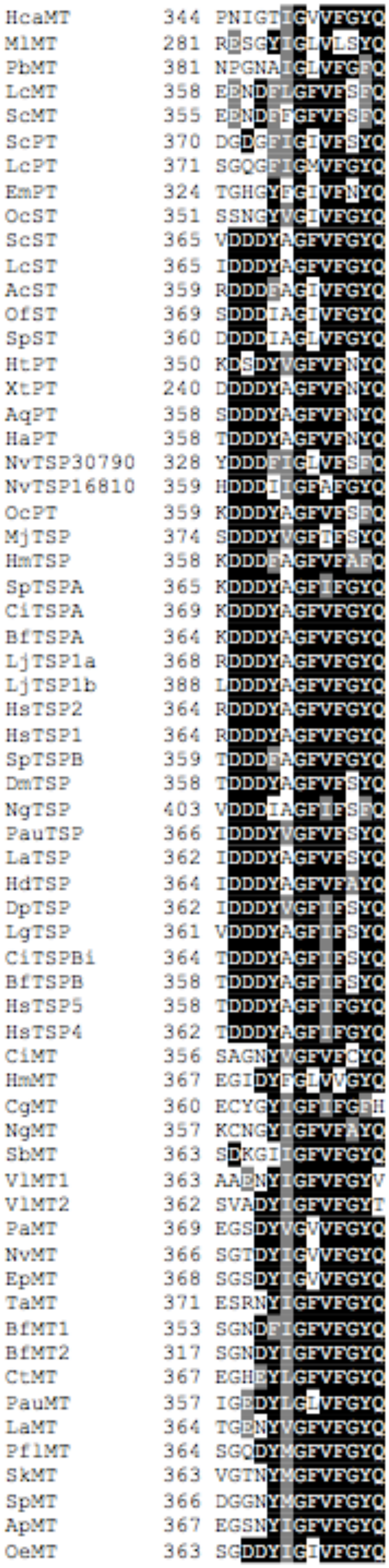

Supplement: Supplementary_Material_msz060 [file supplementary_material_msz060.zip › SupplementaryFig2.pdf]
