# Supplementary figures and images for: Emergence of a Thrombospondin Superfamily at the Origin of Metazoans
Source: Mol Biol Evol. 2019 Mar 13;36(6):1220–38. doi: 10.1093/molbev/msz060 (PMC6526912; doi:10.1093/molbev/msz060)

# Supplementary Figure X

A

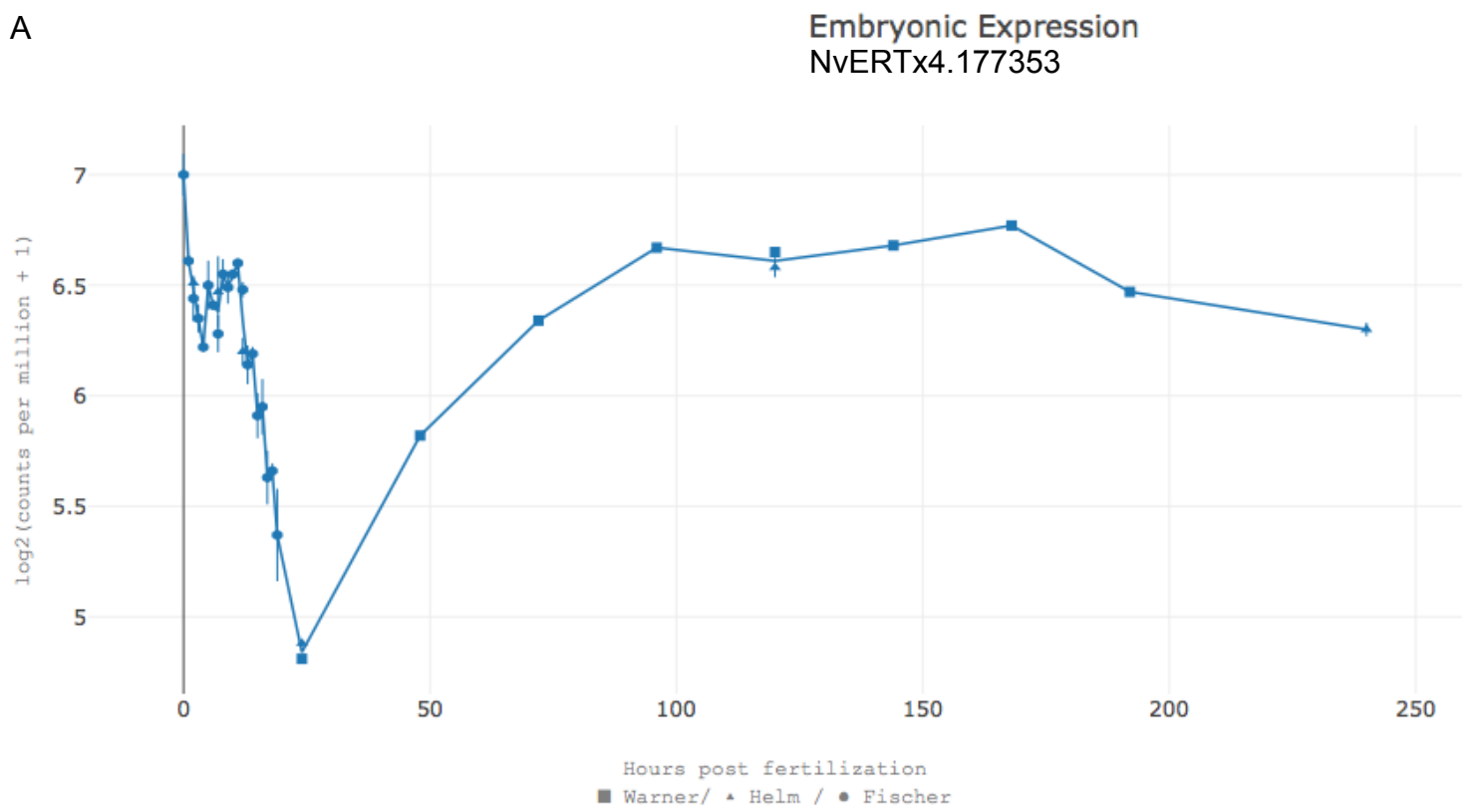

B

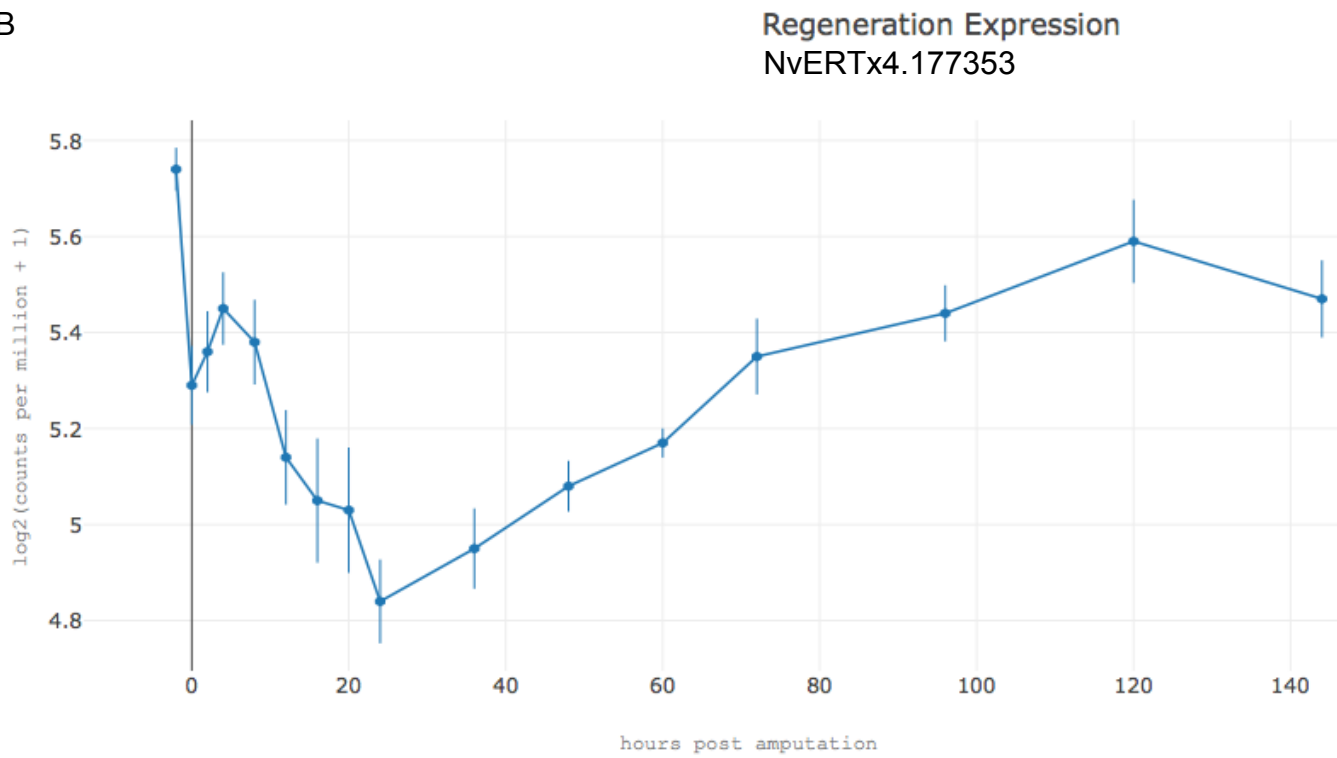

Supplement: Supplementary_Material_msz060 [file supplementary_material_msz060.zip › SupplementaryFig3.pdf]
