# Supplementary material for: Emergence of a Thrombospondin Superfamily at the Origin of Metazoans
Source: Mol Biol Evol. 2019 Mar 13;36(6):1220–38. doi: 10.1093/molbev/msz060 (PMC6526912; doi:10.1093/molbev/msz060)
Supplement: Supplementary_Material_msz060 [file SupplementaryFig4.pdf › SupplementaryFig4.pdf]

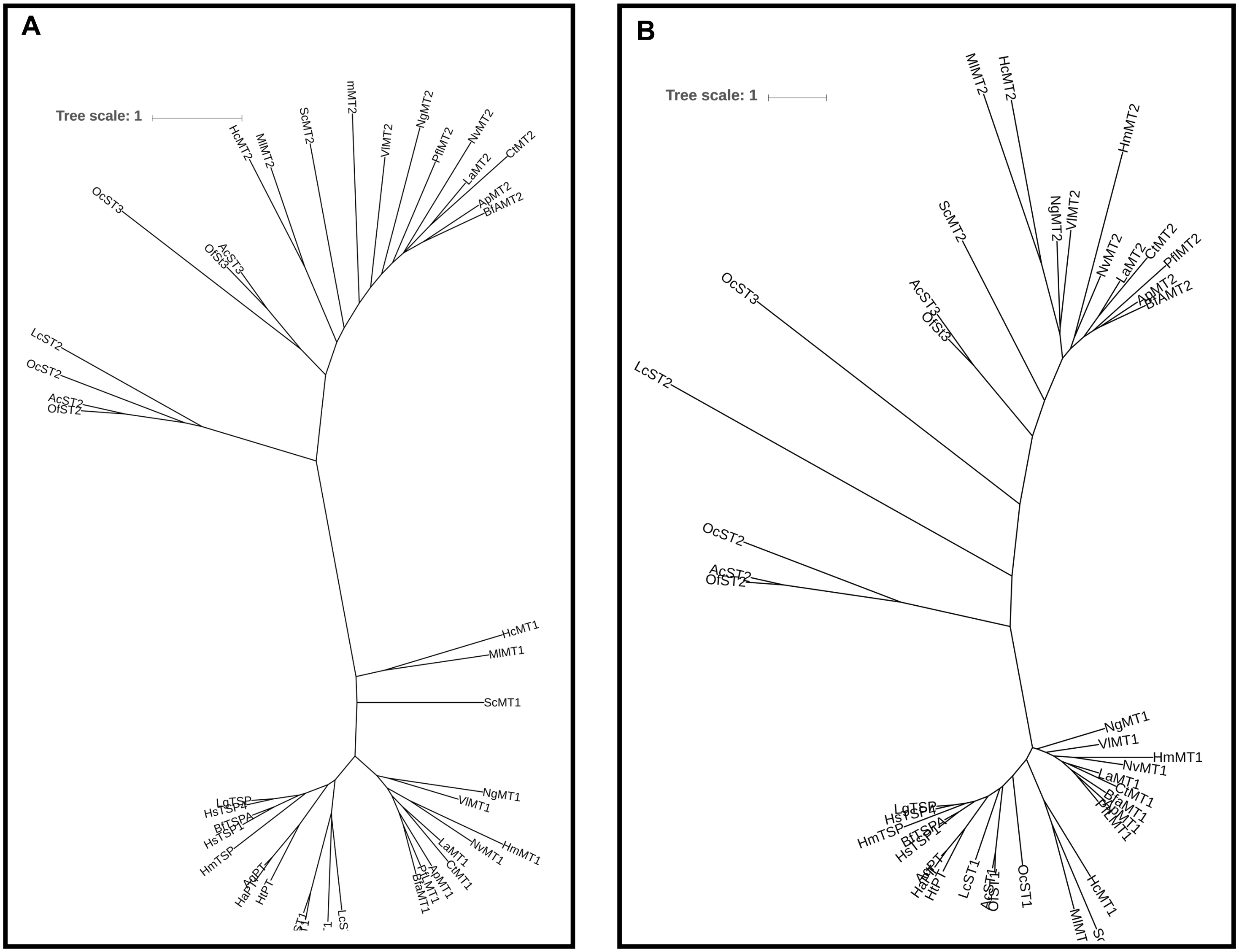

Supplementary Figure 4. Molecular phylogenetic relationships of the Con A-like domains ofThrombospondin Superfamily Members.

The trees are based on individual Con A-like domains (43 sequences and 602 positions) from the indicated mega-TSPs, sushi-TSPs, poriferan-TSPs and TSPs. Sequences were aligned inWebprank (A) or MUSCLE (B) and the trees generated in PhyML with 100 cycles of boot-strapping. For proteins containing more than one Con_A-like domain, the numbering (ST1, ST2, ST3, etc), refers to the first, second or third Con A-like domain, respectively. Key for species as in Table 1.
